# Translational Assessment of Omics Approaches in Endometriosis: Bridging Molecular Discovery with Clinical Utility

**DOI:** 10.3390/ijms27114888

**Published:** 2026-05-28

**Authors:** Ivan Salido-Guadarrama, Oliver Cruz-Orozco, Ignacio Camacho-Arroyo, Juan Carlos Quintero, Jose Roberto Silvestri-Tomassoni, Brenda Sánchez-Ramírez, Mauricio Rodriguez-Dorantes

**Affiliations:** 1 Departamento de Bioinformática y Análisis Estadisticos, Instituto Nacional de Perinatología Isidro Espinosa de los Reyes, Ciudad de México 11000, Mexico; 2 Departamento de Ginecología Quirúrgica, Instituto Nacional de Perinatología, Ciudad de México 11000, Mexico; oliverpco@gmail.com (O.C.-O.); drsilvestri@hotmail.com (J.R.S.-T.); dra.bsr@gmail.com (B.S.-R.); 3 Unidad de Investigación en Reproducción Humana, Instituto Nacional de Perinatología-Facultad de Química, Universidad Nacional Autónoma de México, Ciudad de México 11000, Mexico; ignacio.camacho@inper.gob.mx; 4 Facultad de Química, Universidad Nacional Autónoma de México, Ciudad de México 04510, Mexico; jcchimiste@gmail.com; 5 Instituto Nacional de Medicina Genómica, Periférico Sur 4809, Ciudad de México 14610, Mexico; mrodriguez@inmegen.gob.mx

**Keywords:** endometriosis, multi-omics, biomarkers, clinical translation

## Abstract

Endometriosis affects an estimated 5–10% of women of reproductive age and presents with substantial clinical and biological heterogeneity. Recent clinical guidelines have shifted toward symptom-guided diagnosis supported by expert imaging, moving away from mandatory diagnostic laparoscopy and redefining the evidentiary standards for evaluating new diagnostic technologies. Advances across omics domains, including genomics, epigenomics, transcriptomics, proteomics, metabolomics, extracellular vesicle profiling, microbiome research, and multi-omics integration, have deepened understanding of lesion biology, immune dysregulation, metabolic alterations, and progesterone resistance. However, translation of these molecular insights into clinically actionable tools remains limited. Most candidate biomarkers remain at discovery or internal/developer-led validation stages, constrained by small sample sizes, heterogeneous analytical platforms, incomplete control of confounding variables, and limited independent multicenter validation. In this review, we apply a four-tier evidence-maturity framework, spanning discovery, internal or developer-led validation, independent external validation, and demonstrated clinical utility, to classify omics-based diagnostic, prognostic, and treatment-response applications in endometriosis. We also distinguish potential clinical roles, including triage, adjunctive testing, and replacement-test evaluation, each requiring different validation standards and performance thresholds. Salivary microRNA currently represents the most clinically advanced diagnostic omics candidate, but the available evidence remains developer-led and is best classified as advanced Tier 2/Tier 2+ rather than independent Tier 3 validation. Prognostic and treatment-response applications are less mature and remain discovery-stage because prospective patient-level longitudinal validation and biomarker-stratified treatment trials are lacking. Overall, no omics-derived biomarker has yet achieved independent Tier 3 validation or Tier 4 readiness for routine clinical implementation. At present, omics approaches should be regarded primarily as research and translational prioritization tools rather than determinants of routine clinical decision-making.

## 1. Introduction

Endometriosis is a chronic, estrogen-dependent inflammatory disease characterized by the presence of endometrial-like tissue outside the uterus, affecting approximately 5–10% of women of reproductive age worldwide and representing a major cause of pelvic pain, infertility, and diminished quality of life [1,2,3,4]. The disease exhibits marked clinical and biological heterogeneity, with considerable variation in lesion location, pain perception, mechanisms underlying infertility, comorbidities, and treatment responses [1,5]. This complexity, together with ongoing uncertainty regarding its etiology, continues to hinder timely diagnosis and the development of effective individualized care strategies.

Historically, the gold standard for diagnosis has been laparoscopic visualization with histological confirmation, an approach that is invasive, operator-dependent, and not well suited to early detection or longitudinal monitoring. Conventional management has relied primarily on hormonal suppression and surgery; however, these interventions frequently provide only partial or temporary relief, are associated with side effects, and may not align with patients’ reproductive goals. Recent international guidelines have shifted toward a symptom-driven clinical suspicion supported by high-quality transvaginal ultrasound and magnetic resonance imaging, particularly when performed by experienced operators [4,6,7,8]. As a result, surgical confirmation is no longer required for diagnosis in many patients, thereby redefining the clinical reference standard against which new molecular diagnostic tools must be evaluated.

Concurrently with these changes in clinical practice, advances in omics technologies have transformed research into the pathophysiology of endometriosis. Genome-wide association studies (GWASs) have identified susceptibility loci with modest effect sizes, while transcriptomic, epigenomic, proteomic, and metabolomic analyses have revealed lesion-specific molecular signatures, immune dysregulation, and altered hormonal signaling, including progesterone resistance [1,9,10]. Collectively, this evidence supports the concept that endometriosis comprises a spectrum of biologically distinct disease entities rather than a single uniform condition. Within this framework, precision medicine—defined as the integration of molecular profiles with clinical phenotyping to improve diagnosis, stratification, and treatment selection—has emerged as a prominent research objective, although clinical implementation remains aspirational [9,10,11]. Despite the identification of numerous candidate biomarkers across omics platforms, no omics-derived marker or panel has achieved the level of validation required for routine clinical implementation [11,12,13], largely due to methodological heterogeneity, small sample sizes, inadequate control of confounding variables, and limited external validation [14,15,16].

### 1.1. Unique Contribution of This Review

While previous reviews have provided comprehensive summaries of omics findings across several discovery platforms [17,18], highlighted the methodological challenges intrinsic to non-invasive biomarker validation [13,19], and stressed the aspirational status of precision medicine in endometriosis [9,10], they have not systematically evaluated evidence maturity using explicit translational criteria. Furthermore, prior syntheses have not benchmarked omics candidates against contemporary imaging-based diagnostic pathways or mapped biomarker applications to specific clinical decision points. Table 1 outlines the distinguishing features of the present review in comparison to recent published syntheses.

Building upon these prior contributions, the present review adopts a translational perspective through the use of a four-tier evidence-maturity framework (Box 1), adapted from established biomarker development pathways [3,5,6,7], to classify omics applications from initial discovery through to demonstrated clinical utility. In this context, omics strategies are evaluated against contemporary diagnostic benchmarks, including symptom-driven clinical assessment and expert imaging [8,9,10], rather than historical standards based solely on laparoscopy. Potential clinical roles, namely, triage, adjunctive, and replacement applications, are mapped to specific performance thresholds, and the current maturity of evidence is systematically assessed across omics domains [11,12,13].

Box 1 **Four-tier evidence-maturity framework.**

 Rationale: Biomarker development for clinical implementation typically proceeds in stages, from initial discovery, to progressively more rigorous validation, and finally to demonstration of clinical utility, as outlined in established frameworks from EDRN, IOM, TRIPOD, and STARD [3,5,6,7,11,12]. We adapt these frameworks to classify omics applications across four evidence maturity tiers [13,20,21]. TIER 1: DISCOVERYTIER 2: INTERNAL AND DEVELOPER-LED EXTERNAL VALIDATIONDefinition: Initial identification of candidate biomarkers, molecular signatures, or mechanistic signals through exploratory studies. Operational criteria: Retrospective or cross-sectional designs; small sample sizes, typically *n* < 200; single-center cohorts; case-control comparisons often using healthy controls; hypothesis generation; no independent validation, or only exploratory internal resampling. Clinical role: Hypothesis-generating only. Tier 1 findings can inform disease biology and candidate prioritization, but they do not support clinical decision-making. Methodological anchors: Biomarker-discovery phases and early-phase development principles [3,11,12,13,21].Definition: Evidence beyond discovery, including analytical validation, internal validation, and developer-led external validation. Operational criteria: Prespecified candidate assays or models; standardized analytical workflows; internal validation or developer-led external testing; and, for advanced Tier 2/Tier 2+, prospective, blinded, multicenter evaluation using prespecified procedures and benchmarking against contemporary diagnostic pathways. Clinical role: Research use only. This tier can establish analytical validity and preliminary clinical performance, but it does not by itself demonstrate independent generalizability. Methodological anchors: Analytical validity standards, biomarker-pipeline methodology, and translational omics principles [3,5,11,12,21].TIER 3: INDEPENDENT EXTERNAL VALIDATIONTIER 4: DEMONSTRATED CLINICAL UTILITYDefinition: Rigorous evaluation by research groups independent from biomarker developers, assay owners, or commercial sponsors, in clinically representative populations. Operational criteria: Independent replication by non-developer groups; multicenter enrollment across at least three geographically and clinically distinct sites; prospective or prospectively specified cohort designs with consecutive eligible patients; disease prevalence reflecting real-world settings; head-to-head comparison with relevant standard diagnostic pathways; prespecified analysis plans; and STARD/TRIPOD-adherent reporting. Clinical role: Conditional. Findings at Tier 3 may support consideration for clinical use in defined settings, but clinical utility remains unproven. Methodological anchors: Independent external-validation methodology, diagnostic-accuracy reporting standards, prediction-model validation, and transportability principles [6,7,11,12,13,22,23].Definition: Evidence that biomarker-guided use improves patient-relevant outcomes compared with standard care. Operational criteria: Randomized or otherwise robust comparative studies of biomarker-guided management; patient-centered outcomes such as symptom burden, quality of life, fertility, time to diagnosis, or treatment selection; cost-effectiveness analyses; real-world implementation studies; and guideline incorporation. Clinical role: Established. Supports routine clinical use within the intended-use setting and population in which clinical utility has been demonstrated. Methodological anchors: Biomarker-development clinical utility phases, translational omics implementation principles, and evidence standards requiring improved patient-relevant outcomes [3,5,11,12,21]. TERMINOLOGY NOTE Advanced Tier 2/Tier 2+ is used here as an operational refinement within Tier 2, not as a separate fifth tier. It recognizes that developer-led prospective multicenter validation may provide stronger evidence than single-center discovery or internal validation, particularly for proprietary or commercially developed assays. However, Tier 3 is reserved for independent external validation conducted by investigators not involved in biomarker development, assay ownership, or commercial sponsorship [11,12,13]. For this review, Tier 3 requires validation across at least three geographically and clinically distinct sites as a pragmatic minimum for assessing transportability; this threshold should be interpreted together with independence, clinical-spectrum diversity, comparator quality, blinding, and prespecified analysis rather than as a universal numerical rule [11,12,13,22,23]. For example, GWAS “replication” demonstrates robust population-level associations but not clinical validity for individual prediction; such findings remain Tier 1 for clinical applications [11,16,20]. Abbreviations: EDRN: Early Detection Research Network, IOM: Institute of Medicine (now known as the National Academy of Medicine), TRIPOD: Transparent Reporting of a multivariable prediction model for Individual Prognosis Or Diagnosis, STARD: Standards for Reporting Diagnostic Accuracy Studies.

For clarity and consistent application, Box 1 presents each evidence-maturity tier using a common structure: definition, operational criteria, clinical role, and methodological anchors. These anchors link the framework to established biomarker-development, diagnostic-reporting, external-validation, transportability, and clinical utility principles [3,5,6,7,14,16,20,23].

### 1.2. Approach to Literature Search and Synthesis

This article is a structured narrative review and translational evidence-maturity assessment, not a de novo systematic review. We therefore did not conduct PRISMA-style dual screening, risk-of-bias assessment for every individual study, or quantitative meta-analysis. To improve transparency, we constructed and verified the evidence base using a modular PubMed/MEDLINE search strategy, supplemented by targeted searching and reference list screening.

The primary disease-specific search was conducted in PubMed/MEDLINE for English-language, human-study publications from January 2014 through December 2025, with preprints excluded from the primary search set. The search was run and verified during revision in May 2026. Five modular search blocks combined endometriosis terms with domain-specific terms:

1. Omics and biomarkers, combining endometriosis with biomarker, diagnosis, prognosis, treatment response, and omics-domain terms, including genomics, transcriptomics, proteomics, metabolomics, microRNA, miRNA, and multi-omics;
2. Epigenomics and genetic susceptibility, combining endometriosis with epigenomics, DNA methylation, GWASs, genome-wide association, and polygenic risk score;
3. Microbiome and extracellular vesicles, combining endometriosis with microbiome, microbiota, extracellular vesicles, exosomes, and liquid biopsy;
4. Artificial intelligence and multi-omics integration, combining endometriosis with artificial intelligence, machine learning, deep learning, multi-omics integration, diagnosis, prediction, and classification;
5. Imaging and diagnostic pathways, combining endometriosis with imaging, ultrasound, transvaginal ultrasound (TVUS), MRI, diagnostic accuracy, systematic review, guideline, and Cochrane terms.

Methodological standards for biomarker development, external validation, diagnostic-accuracy reporting, and prediction-model reporting, including EDRN, STARD, TRIPOD, and diagnostic-accuracy sample-size methodology, were identified through targeted author- and title-based searches and reference list screening, because these papers are not endometriosis-specific and are not reliably captured by disease-focused search strings.

We prioritized (i) clinical guidelines and consensus documents for current diagnostic pathways and benchmark comparators; (ii) systematic reviews and meta-analyses for domain-level evidence summaries; (iii) large prospective, multicenter, or externally validated studies for candidate biomarker performance; and (iv) methodological standards relevant to biomarker development, diagnostic accuracy, and clinical utility. Tier assignments were restricted to human clinical studies reporting diagnostic, prognostic, or treatment-response outcomes. Preclinical, animal, and mechanistic lesion-based studies were used to contextualize biological plausibility but did not determine evidence-maturity classification.

The search was supplemented by manual screening of reference lists from retrieved systematic reviews, clinical guidelines, and key primary studies. Foundational disease reviews, clinical-context papers, and non-endometriosis-specific references that provide essential background were identified through reference list scanning and the authors’ domain expertise. The narrative approach was guided by quality principles for non-systematic reviews [24].

## 2. Clinical Landscape: Reference Standards and Unmet Needs

### 2.1. Clinical Heterogeneity and Disease Complexity

Endometriosis encompasses a spectrum of anatomical phenotypes, superficial peritoneal disease (SUP), ovarian endometriomas (OMA), and deep infiltrating endometriosis (DIE) [25,26,27]. Conventional classifications, including the revised American Society for Reproductive Medicine (rASRM) system, correlate only weakly with pain severity, functional impairment, or fertility outcomes [28,29,30]. Pain phenotypes vary widely and severe pelvic pain may occur in patients with minimal visible disease, while extensive adhesions may be associated with minimal symptoms. These phenotypic differences complicate diagnosis and contribute to diagnostic delays and frequent misdiagnosis.

Infertility affects approximately 30–50% of women with endometriosis [31,32,33,34], resulting from anatomical distortion, a pro-inflammatory peritoneal environment, and defects in endometrial receptivity [29,32]. Disease progression remains difficult to predict, with progression occurring in an estimated 50% of cases. Some women experience stable symptoms, while others develop worsening pain, expanding lesions, or recurrent disease following treatment [29,35,36]. This clinical and biological heterogeneity underpins the rationale for precision-medicine approaches, although these remain aspirational strategies.

### 2.2. Contemporary Diagnostic Pathways

The diagnostic paradigm has substantially evolved. Historically, laparoscopic visualization with histological confirmation was deemed the gold standard, but this approach is invasive and costly, and carries surgical risks [37,38,39]. International guidelines have shifted toward symptom-driven clinical suspicion supported by non-invasive imaging, reserving surgery for patients in whom imaging is inconclusive, symptoms are refractory, or fertility intervention is planned [8,9,35].

To clarify the real-world benchmark that molecular tests must plausibly match or improve against, Table 2 summarizes diagnostic performance estimates for contemporary expert imaging across major endometriosis phenotypes and clinical settings. Transvaginal ultrasound (TVUS) is recommended as first-line imaging and shows the strongest diagnostic performance for ovarian endometrioma (OMA) and deep infiltrating endometriosis (DIE) when performed by experienced operators who adhere to standardized protocols [40,41]. However, TVUS performance is operator-dependent and is still limited for superficial peritoneal disease [42,43]. Magnetic resonance imaging (MRI) complements TVUS by improving soft-tissue characterization and mapping of DIE, particularly when bowel, bladder, or ureteral involvement is suspected [35,37,41]. Taken together, this diagnostic context supports the benchmarking of proposed molecular tests against symptom-driven assessment integrated with expert imaging rather than against historical laparoscopy-only standards [42,43].

### 2.3. Clinical Challenges and Unmet Needs

Persistent diagnostic delay: Average diagnostic delays of 6–12 years remain common, driven by non-specific symptoms that overlap with other conditions [34,36,48], normalization of pain [43], and continued dependence on laparoscopy for early or superficial disease [35]. Even expert imaging can fail to detect superficial peritoneal lesions and some extrapelvic sites (Table 2), and Cochrane reviews confirm that no imaging or biomarker strategy achieves surgical replacement accuracy spanning all phenotypes [49,50]. This creates a clear role for rigorously validating non-invasive triage tools, rather than wholesale replacement of imaging or surgery.

Weak alignment between diagnosis and prognosis: Surgical staging and lesion phenotype correlate only weakly with pain severity, functional impairment, or fertility outcomes [32,38,51]. Imaging also provides limited insight into molecular features such as progesterone resistance, immune dysregulation, and fibrosis that are likely to drive pain persistence, recurrence, and treatment failure [1,28,52].

Empirical treatment selection: Management is predominantly empirical, with hormonal therapies and surgery prescribed in the absence of biomarkers to predict response, identify progesterone-resistant disease, or guide the timing and extent of surgical intervention [53,54]. This trial-and-error approach contributes to a more prolonged symptom burden, repeated surgeries, and delayed access to effective treatments. Conventional imaging, which primarily reflects morphological features, does not capture these aspects [37,42].

Collectively, these unmet needs suggest three realistic clinical roles for omics tools: (1) triage tests in settings lacking expert imaging, where very high sensitivity (≥95%) and high negative predictive value are required to rule out disease [8,37,42]; (2) adjunctive tests that provide additional information beyond TVUS/MRI in imaging-ambiguous or SUP-predominant disease, necessitating balanced performance [55]; and (3) aspirational replacement tests for laparoscopy, which must meet established Cochrane-derived thresholds of sensitivity ≥ 94% and specificity ≥ 79% [49,50]. These role- and threshold-specific expectations form the basis of the four-tier evidence-maturity framework in Box 1 and guide the subsequent translational assessment.

## 3. Translational Status of Omics Approaches

### 3.1. Diagnostic Applications

**Figure 1:**
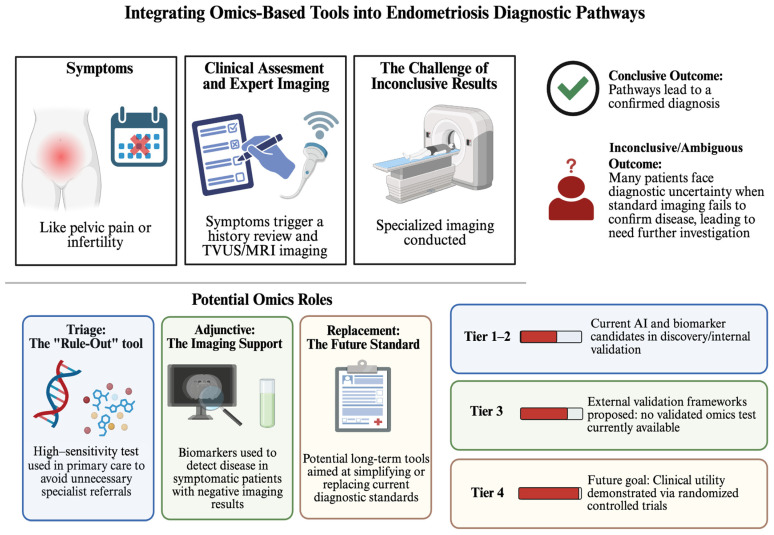
Integrating omics-based diagnostic tools into contemporary endometriosis diagnostic pathways. Current diagnostic standards emphasize symptom-based clinical assessment followed by expert imaging (TVUS/MRI), with surgery no longer mandatory for diagnosis in many cases [8,9]. This vignette highlights how omics-based biomarkers could theoretically contribute at three points: (1) Triage (“rule-out”) tests, prioritizing high sensitivity (≥95%) to identify patients suitable for empirical management versus those requiring specialist referral, particularly relevant in which expert imaging is not readily available; (2) Adjunctive tests, prioritizing high specificity to complement imaging by augmenting detection when disease is not visualized on TVUS/MRI or by fine-tuning risk stratification in imaging-ambiguous cases; and (3) Replacement tests, requiring both sensitivity ≥ 94% and specificity ≥ 79% (Cochrane thresholds; [49,50]) to substitute for diagnostic laparoscopy, which is still a longer-term objective. At present, omics candidates largely remain at Tier 1–2; salivary miRNA (advanced Tier 2/Tier 2+) is closest to clinical translation but requires independent Tier 3 validation prior to implementation. See Section 5.2 for a worked example of a Tier 3 validation design.

Noninvasive diagnostic biomarkers are being developed to reduce diagnostic delay, support triage of symptomatic patients for imaging or surgery, and, more ambitiously, to replace diagnostic laparoscopy in selected phenotypes [2,4,50,56,57]. The potential clinical roles of omics-based diagnostics should be assessed against contemporary imaging performance for OMA and DIE, where qualified TVUS/MRI achieve sensitivities of approximately 0.79–0.95 and specificities of 0.91–0.96, rather than against laparoscopy alone [44,49,58,59]. Current European Society of Human Reproduction and Embryology (ESHRE) guidelines underscore imaging as the mainstay of diagnosis and do not recommend blood or tissue biomarkers for routine clinical use [9,37,60]. Within this context, three pragmatic clinical roles can be distinguished (see Figure 1).

*Triage tests:* Identify patients who can be managed with provisional hormonal therapy and follow-up versus those requiring specialist referral and more advanced imaging. In this setting, very high sensitivity (≥95%) and high negative predictive value are needed to minimize missed disease; moderate specificity (≥50%), although developed in surgery—candidate populations, may be acceptable in primary care settings when expert imaging is unavailable [49].

*Adjunctive tests:* Complement TVUS/MRI by increasing detection of disease that is poorly visualized (e.g., superficial or early disease) or by sharpening risk stratification in imaging-ambiguous cases. Based on experience from multi-marker panels and AI-based diagnostic models, a clinically useful adjunct would be expected to achieve broadly balanced sensitivity and specificity (both ≥ 80%) in representative symptomatic cohorts [17,34,61,62,63].

*Replacement tests:* Substitute for diagnostic laparoscopy in patients with negative or inconclusive imaging for whom surgery would otherwise be clinically considered. Cochrane-derived benchmarks and subsequent biomarker reviews suggest that such tests should reach sensitivity ≥ 94% and specificity ≥ 79% in symptomatic populations to be considered credible alternatives to diagnostic surgery [49,57]. These thresholds are retained here only for surgical replacement claims; they are not intended as universal benchmarks for triage or adjunctive diagnostic roles, where the relevant comparator is the contemporary pathway of symptom-guided assessment supported by expert TVUS/MRI [8,9,35]. Despite promising performance of some proteomic, metabolomic, and miRNA-based signatures, often reporting AUCs above 0.9 in internal or developer-led validation [17,34,61,62,64,65,66,67], no omics-based biomarker panel has met surgical replacement thresholds in large, independent, multicenter studies with robust control of phenotype, menstrual cycle phase, and treatment status. The possibility of achieving true replacement-test performance therefore remains uncertain and would require substantially stronger validation than is currently available [4,49,57,68].

*Cross-domain constraints:* The main barriers to higher-tier diagnostic translation are broadly consistent and often relate to study design and external validity rather than a clear lack of biological signals. The corresponding evidence-maturity placements for diagnostic applications are summarized in Table 3. Frequent issues include small single-center cohorts; two-gate case-control designs that can inflate apparent performance by excluding symptomatic controls; incomplete control of menstrual cycle phase and hormonal treatment; heterogeneous phenotype composition (OMA, DIE, SUP) and variable surgical reference standards; and limited independent, multicenter external validation, particularly head-to-head comparisons against optimized expert imaging in clinically relevant populations [17,34,49,57,63,66,69]. The domain summaries below therefore emphasize what has been studied, performance in context, domain-specific hurdles, and the validation steps most likely to establish role-appropriate clinical utility.

#### 3.1.1. Genomics: Polygenic Risk Scores

*Core idea and specimens:* GWASs and large multi-ancestry meta-analyses have systematically assessed common genetic variation in endometriosis [14,70,72], identifying replicated risk loci near genes involved in sex-steroid signaling, extracellular matrix remodeling, inflammation, and tissue development. A major 2023 meta-analysis included 60,674 cases and 701,926 controls of predominantly European and East Asian ancestry [14] and a subsequent peer-reviewed multi-ancestry GWASs expanded the evidence base to approximately 1.4 million women [71]. These loci have been aggregated into polygenic risk scores (PRSs) using genome-wide significant variants, with some approaches extending to sub-threshold variants [72,99,100].

*Performance in context:* Despite strong statistical replication, endometriosis PRSs explain only about 2–5% of endometriosis variance in current meta-analyses [14,16,70] and show modest discriminatory performance in independent cohorts [71]. This remains far below thresholds needed for clinical screening, triage, or diagnosis [8,49].

*Domain-specific hurdles:* Limited predictive performance reveals a highly polygenic architecture with many variants of tiny effect [16,72], and the fact that GWAS signals primarily reflect population-level risk rather than calibrated individual diagnostic probabilities [15,71,72]. Most loci are non-coding regulatory alleles with incompletely defined functional consequences, and GWASs do not capture gene-environment interactions or other contributors to risk, including epigenetic and microbiome-related influences [100].

*Translational bridge:* In the near term, PRSs are most defensible as mechanistic tools for disease biology and target nomination (including shared mechanisms with pain and inflammatory comorbidities), rather than as actionable diagnostics; role-expansion would require demonstration of calibrated individual risk prediction with clinically meaningful decision impact in representative care settings [100,101,102].

#### 3.1.2. Epigenomics

*Core idea and specimens:* Epigenomic studies, most commonly DNA methylation profiling using Illumina 450K and EPIC arrays, have compared ectopic lesions with eutopic endometrium from women with and without endometriosis, with some work also examining blood-based signals [21,68,73,74]. Reported differentially methylated regions implicate steroid hormone signaling (ESR1, PGR), immune regulation (HLA loci, cytokine receptors), and tissue remodeling pathways (MMPs, TIMPs) [68,74,103,104]. Epigenomic studies also implicate altered chromatin regulation and enhancer activity in pathways related to progesterone resistance, inflammation, and fibrotic remodeling [54,104,105].

*Performance in context:* Although epigenomic alterations are repeatedly observed and biologically plausible, reported blood-based methylation effects are typically small and overlap across studies is limited, and proposed methylation classifiers have not yet been validated in clinically representative cohorts. No epigenomic panel has been validated for diagnosis, prognosis, or treatment-response prediction in applied clinical settings [1,2].

*Domain-specific hurdles:* Consistent with cross-domain constraints, most epigenomic studies remain small (typically 20–80 patients), cross-sectional, and focused on surgically obtained lesion or eutopic tissue rather than accessible biospecimens, limiting diagnostic applicability [21,22,65,68]. Reproducibility is hampered by cell-type heterogeneity, inconsistent control of menstrual cycle phase and hormonal treatment, and variation in platforms and analytic pipelines [68,74]. While some AI-derived methylomic models report promising internal accuracy, low signal-to-noise ratios and lack of independent, prospective external validation currently preclude clinical interpretation [63].

*Translational bridge:* Epigenomic profiling is most defensible today as a mechanistic layer that clarifies transcriptional dysregulation and progesterone resistance, rather than as a clinical diagnostic or prognostic tool [54,75]. Movement toward clinical use would require analytically robust signatures from accessible biospecimens, standardized pre-analytics with explicit control of hormonal and cycle effects, and independent prospective validation in well-phenotyped cohorts.

#### 3.1.3. Lesion-Based Transcriptomics

*Core idea and specimens:* Lesion-based transcriptomics profiles gene expression in ectopic lesions versus eutopic endometrium using microarrays and RNA sequencing, focused on mapping disease biology and nominating signatures [1,20]. Across systematic reviews, recurrently implicated pathways include inflammation (IL1B, TNF, PTGS2), steroid metabolism (CYP19A1, HSD17B1), extracellular matrix remodeling (MMPs, integrins), angiogenesis (VEGF, FGF2), and progesterone signaling (PGR isoforms, FOXO1) [1,2]. Some studies have proposed molecular subtypes from transcriptomic clustering, such as stroma-enriched and immune-enriched profiles [106,107].

*Performance in context:* While many studies report differential expression and pathway-level convergence, proposed transcriptomic subtypes generally show minimal reproducibility across independent cohorts [1,58,76]. In practice, these data have been more consistent as descriptive biology than as stable, transportable signatures for clinical prediction [108].

*Domain-specific hurdles:* Consistent with cross-domain constraints, lesion-based studies usually rely on small surgical cohorts (median *n* ≈ 20–50), with heterogeneous inclusion of peritoneal, OMA, and DIE phenotypes, inconsistent documentation of menstrual cycle phase, and varied platforms and analytical pipelines [2,76,77]. Cross-study overlap in differentially expressed genes is often low, plausibly reflecting biological heterogeneity, batch effects, and non-standardized tissue collection and processing [106,108]. Because these signatures require surgically obtained tissue, they are inherently unsuitable as noninvasive diagnostic tools, and no lesion-based transcriptomic panel has been prospectively validated for diagnosis, prognosis, or treatment-response prediction.

*Translational bridge:* The most realistic near-term contribution is mechanistic and hypothesis-generating: pathway nomination and candidate molecular subtyping which might inform future stratified trial design [107,109]. Any progression toward clinical prediction would require standardized tissue-processing workflows and prospective validation linked to clinically relevant outcomes beyond discovery-only differential expression.

#### 3.1.4. Blood-Based Transcriptomics

*Core idea and specimens:* Blood-based transcriptomic approaches measure circulating miRNAs, mRNAs, and other RNA species to create non-invasive signatures intended for diagnostic triage or adjunctive testing [66,77,79,80]. Panels typically include 5–15 miRNAs selected from small discovery cohorts, and reported candidates include miR-125b-5p, miR-150-5p, miR-342-3p, miR-451a and miR-30c-5p [66,80,110,111]. Some case-control studies report moderate and variable discrimination (AUC 0.60–0.9) and related work has explored circulating mRNA signatures and lncRNAs at earlier discovery stages [34,109,112,113].

*Performance in context:* Reported diagnostic performance is variable and frequently derived from designs that can overestimate accuracy, and replication of specific miRNA signals is inconsistent when markers are tested in new cohorts [66,81,106]. Where independent evaluation exists, effect sizes frequently attenuate or cannot be replicated [57,114].

*Domain-specific hurdles:* Methodical constraints dominate this literature. Most studies enroll 30–100 women per group [66,79,80,108] and use two-gate case-control designs comparing laparoscopy-confirmed cases to healthy controls, which can inflate diagnostic performance by excluding symptomatic but endometriosis-negative controls. Menstrual cycle phase is often not reported, despite evidence that circulating miRNA profiles vary across the cycle and hormonal changes [23,66,83,111]. Pre-analytical factors, including hemolysis, platelet contamination, and non-standardized extraction and normalization protocols, add to the complexity of cross-study comparability and likely contribute to modest reproducibility [66,87,106].

*Translational bridge:* Current utility is best framed as research-stage signal generation and biology discovery. Clinical progression toward triage or adjunctive testing would require harmonized pre-analytics, cycle-standardized sampling, inclusion of symptomatic controls, and independent prospective validation with head-to-head benchmarking against imaging pathways [1,81,106].

#### 3.1.5. Salivary microRNA Signatures

*Core idea and specimens:* Salivary microRNA (miRNA) profiling currently represents the most clinically advanced omics-based diagnostic candidate reported in endometriosis, based on developer-led prospective multicenter validation of a 109-miRNA classifier [65,66,78]. Bendifallah and colleagues developed the ENDOmiRNA/ENDOTEST saliva-based signature using genome-wide small RNA sequencing, machine-learning feature selection, internal cross-validation, and subsequent prospective multicenter validation [65,84,114]. In the full validation cohort, the test was evaluated in 971 symptomatic patients recruited across 17 French centers and showed high diagnostic accuracy for binary detection of endometriosis. Endometriosis was diagnosed by imaging, laparoscopy, or both, while control patients underwent laparoscopy [65,84]. Saliva samples were collected irrespective of menstrual cycle phase and hormonal or analgesic treatment, and hormonally treated participants were included.

*Performance in context:* In the multicenter validation cohort, the salivary miRNA classifier demonstrated high diagnostic accuracy for detecting endometriosis, achieving a sensitivity of 97.3% (95% CI 96.4–98.0%), a specificity of 94.1% (95% CI 91.0–96.4%), and an overall diagnostic accuracy of 96.6% [84]. We used these estimates to project predictive values at lower prevalence to illustrate the effect of the clinical spectrum. For instance, at 30% prevalence, the projected positive predictive value (PPV) is approximately 87.6% and the negative predictive value (NPV) is approximately 98.8%; at 40% prevalence, the projected PPV is approximately 91.7% and the NPV is approximately 98.1%. These predictive values were calculated using the standard relationships among prevalence, sensitivity, specificity, PPV, and NPV. However, such projections assume constant performance across populations, which may not hold true due to potential variation in disease spectrum, clinical setting, comparator pathway, and phenotype distribution. Consequently, direct validation in lower-prevalence symptomatic populations is required before these projections can inform clinical implementation.

*Why this remains advanced Tier 2/Tier 2+ rather than Tier 3 or Tier 4:* Despite being the most advanced omics-based diagnostic candidate in endometriosis, the current evidence remains best classified as advanced Tier 2/Tier 2+ rather than Tier 3 or Tier 4. The available validation is prospective and multicenter, but it remains developer-led, uses a proprietary classifier/platform, and has not yet been independently replicated by a non-developer group [65,84]. All validation centers were located in France, so geographic and health-system transportability remain unproven. In addition, clinical utility in the sense of improved patient management, patient-relevant outcomes, cost-effectiveness, or guideline-level implementation has not yet been demonstrated.

*Diagnostic target and role limitation:* The assay should be interpreted as a binary detection tool for endometriosis diagnosed by imaging, laparoscopy, or both, rather than as a subtype-specific diagnostic replacement for expert imaging. Although subgroup analyses have been reported for surgically confirmed disease, rASRM I–II disease, and complex diagnostic cases, the test has not been validated as a replacement for imaging-based anatomical characterization of ovarian endometrioma or deep infiltrating endometriosis [65,84]. Its most plausible near-term role is therefore triage or adjunctive risk stratification, particularly if independent validation confirms performance in lower-prevalence symptomatic populations and in settings where expert imaging access is variable.

*Hormonal treatment and cycle-phase interpretation:* The inclusion of hormonally treated participants and sampling irrespective of menstrual cycle phase are clinically relevant strengths, because many symptomatic patients are already receiving treatment when diagnostic uncertainty arises [83]. In the full validation cohort, saliva samples were collected regardless of menstrual cycle phase and hormonal or analgesic treatment, and hormonally treated participants were included [65,84]. However, because separate treated-versus-untreated sensitivity and specificity estimates were not available from the documents reviewed, their reported lack of apparent hormonal-treatment influence should be interpreted cautiously and confirmed in future independent validation studies.

*Translational and regulatory context:* Within the original French validation and health-technology-assessment context, conditional reimbursement and clinical utility evaluation processes have begun to define the possible clinical positioning of the salivary miRNA test (ClinicalTrials.gov: NCT06794424). These developments support the translational relevance of the candidate but do not constitute Tier 4 evidence, because improved patient management, patient-relevant outcomes, cost-effectiveness, and guideline-level implementation remain to be demonstrated.

#### 3.1.6. Proteomics

*Core idea and specimens:* Proteomic studies have evaluated plasma, serum, peritoneal fluid, and menstrual effluent, typically using mass spectrometry discovery followed by targeted validation with ELISA or multiplex immunoassays [2,17,62,78,85]. Frequently studied candidates include CA-125, glycodelin, VEGF, annexin V, and osteopontin, as well as multi-protein panels combining these signals. A commonly cited “best-developed” example is a four-protein plasma panel (annexin V, VEGF, CA-125, and glycodelin or sICAM-1) evaluated in a single-center prospective laparoscopy cohort, reporting sensitivity of 81–90% and specificity of 63–81% for ultrasound-negative endometriosis [61].

*Performance in context:* Across systematic reviews and meta-analyses, CA-125 alone shows insufficient sensitivity (~40–60%) despite moderate-to-high specificity (~70–93%), performs better in stage III/IV than in stage I/II disease, and overlaps substantially with other benign gynecologic conditions, menstruation, and pregnancy [62]. ESHRE guidelines therefore do not support CA-125 as a standalone diagnostic test [9,62,115]. Multi-protein panels, including inflammatory and angiogenic combinations, generally offer only modest gains over CA-125 and remain below accuracy thresholds needed for routine implementation [4,34,116]. Although some recent ten-protein models report very high AUCs (up to 0.997), these are derived from highly selected populations and have not been tested in broader, lower-prevalence clinical settings. Even the Vodolazkaia four-protein panel, despite prospective design and internal test-set validation, showed lower performance when re-evaluated in subsequent cohorts by the same group [17,61,85].

*Domain-specific hurdles:* Much of the proteomic literature remains discovery-heavy, with small cohorts (often *n* < 100), heterogeneous inclusion criteria and cycle-phase timing, and highly divergent lists of differentially expressed proteins [17,86]. A systematic review identified 644 differentially expressed proteins across non-invasive samples, with sensitivity (38–100%) and specificity (59–99%) ranges that indicate substantial between-study heterogeneity [17]. Cross-study overlap is minimal, consistent with pre-analytical variability, platform differences, and underlying biological heterogeneity [17,87].

*Translational bridge:* Most individual proteins and small panels remain Tier 1, while the Vodolazkaia four-protein panel and similar internally validated plasma models fit lower Tier 2, given prospective single-center evaluation without independent multicenter replication [86,87]. Progression toward clinical triage or adjunctive use would require standardized pre-analytics, independent multicenter validation in representative symptomatic cohorts, and explicit benchmarking against imaging-based diagnostic pathways.

#### 3.1.7. Metabolomics

*Core idea and specimens:* Metabolomic profiling has evaluated urine, serum, plasma, and peritoneal fluid using nuclear magnetic resonance (NMR) spectroscopy and mass spectrometry coupled with liquid or gas chromatography (LC-MS, GC-MS) [18,88,117,118]. Reported signals commonly involve lipid metabolism, including phospholipids, sphingolipids and fatty acids, amino acid pathways such as tryptophan and phenylalanine metabolism, and energy or oxidative stress-related metabolites, including Krebs cycle intermediates [18,118,119]. Candidate panels vary widely, from small metabolite sets to panels of 30 or more metabolites, and some discovery studies report high discrimination (AUC 0.75–0.99).

*Performance in context:* Systematic reviews show substantial heterogeneity in identified metabolites, with minimal overlap across studies, with <15% replication reported, which limits confidence that current panels are transportable across cohorts or platforms [117,120]. Most evidence derives from small, single-center, surgically defined case-control comparisons (*n* = 30–100), and no metabolomic signature has undergone external validation in independent prospective cohorts.

*Domain-specific hurdles:* Between-study inconsistency reflects variation in sample type (urine vs. serum vs. peritoneal fluid), analytic platforms (NMR vs. LC-MS vs. GC-MS), pre-analytical handling, and metabolite annotation methods [4,18,120]. Metabolite levels are also sensitive to diet, circadian rhythms, medication use, and sample handling, confounders that are not consistently measured or controlled in many studies [18]. Several reported signals appear phenotype-specific, such as profiles that distinguish OMA from DIE, which may further limit generalizability across the disease spectrum [18,34,88].

*Translational bridge:* Moving toward clinical translation will likely benefit from greater standardization of protocols and annotation pipelines, larger and better-phenotyped cohorts with systematic confounder assessment, and independent external validation across varied types of phenotypes and care settings, to more confidently define the diagnostic utility of metabolomic signatures [34].

#### 3.1.8. Extracellular Vesicles

*Core idea and specimens:* Extracellular vesicles (EVs), including exosomes, are bioactive nanovesicles of roughly 30 to 150 nm released via exocytosis that carry RNAs, proteins, lipids, and small non-coding RNAs [90,121]. Studies have profiled EV cargo, including miRNAs, lncRNAs, and proteins, reporting differential patterns in women with endometriosis versus healthy, fertile controls, alongside mechanistic hypotheses around EV-mediated communication participating in lesion establishment, inflammation, and angiogenesis [90,121,122,123].

*Performance in context:* EV research in endometriosis remains primarily early-stage and exploratory, with most evidence derived from small, single-center studies (typically *n* = 5–50 per group) [123]. As a result, candidate EV signatures have not yet reached the level of reproducibility or external validation needed to support diagnostic translation, and no EV-based panel has been validated in large, independent, multicenter cohorts or compared head-to-head with simpler biomarker approaches [121].

*Domain-specific hurdles:* EV isolation and characterization methods vary substantially, including ultracentrifugation, size-exclusion chromatography (SEC), and commercial precipitation kits, which can yield different EV populations with variable purity and downstream “signature” stability [121,123,124]. Although high-quality studies adhere to the Minimal Information for Studies of Extracellular Vesicles (MISEV) guidelines, consistency in compliance and nomenclature remains uneven across the literature [123,124]. Contamination with non-EV particles such as lipoproteins and protein aggregates is a recognized technical limitation, including cases where nanoparticle tracking analysis detects particles that are not visualized by electron microscopy [125]. In parallel, the functional significance of differential EV cargo in vivo remains partly established, which complicates interpretation of observed associations and prioritization of candidates for validation [121,123,124].

*Translational bridge:* A positive path forward would emphasize harmonized EV isolation and reporting standards with explicit purity controls, demonstration of analytical validity, and stepwise validation in representative cohorts, including direct comparisons against simpler biomarker approaches to clarify whether EV-based markers offer incremental clinical value.

#### 3.1.9. Gut Microbiome

*Core idea and specimens:* Gut microbiome studies have compared microbial composition in women with endometriosis versus controls using 16S rRNA gene sequencing and shotgun metagenomic sequencing [91,126,127]. Reported signals include differences in alpha and beta diversity and changes in the relative abundance of genera such as Prevotella, Blautia, and Gardnerella, even though specific findings vary across studies [92,93,127,128,129]. Mechanistic hypotheses link microbiome alterations to immune dysregulation, estrogen metabolism via bacterial beta-glucuronidase activity, and gut barrier dysfunction [19,91,93,130,131].

*Performance in context:* Across the current literature, reproducible microbiome signatures suitable for diagnosis have not emerged, and associations reported in individual cohorts often do not generalize across studies [92,128,132]. Consistent with this, no validated diagnostic signature is currently available.

*Domain-specific hurdles:* Gut microbiome composition is strongly affected by geography, diet, antibiotics, hormonal contraceptives, menstrual cycle phase, body mass index, and comorbidities, and these factors are not consistently measured or controlled in many studies [128,132]. Reported associations are often at the genus or family level, with limited consistency at the species level across cohorts [127,133,134]. Many studies also remain small (typically *n* = 30–100 per group), single-center, and retrospective, heightening susceptibility to geographic bias and confounding [19,91]. Finally, mechanistic interpretations remain provisional, because current human data only limitedly distinguish causal contributions from consequence of disease, shared risk factors, or treatment-related effects [126,129].

*Translational bridge:* A microbiome-specific route to stronger inference is to treat exposure control and technical variation as first-order design variables: tightly document or standardize diet and medication, especially antibiotics and hormonal contraception, align extraction and sequencing workflows to reduce batch-driven signals, and use repeated or longitudinal sampling to account for within-person temporal variability and clarify directionality [91,128,132].

### 3.2. Prognosis and Patient Stratification

Precision medicine in endometriosis requires tools that can stratify patients by likely clinical course, for example, persistent or progressive pain, recurrence risk, treatment resistance, or infertility-related complications [26,32,43]. In contrast, current surgical staging and classification systems, including rASRM, correlate only weakly with symptom severity, treatment response, and future reproductive outcomes, which motivates interest in biology-based stratification that links molecular profiles to clinically meaningful phenotypes [26,31,33,77,98]. Plausible molecular candidates exist across multiple omics domains; what is absent is longitudinal, patient-level validation demonstrating that any profile predicts outcomes over time. Consequently, all prognostic applications currently remain Tier 1 (see Table 4 for details).

Molecular subtyping: Multiple studies have proposed transcriptomic, proteomic, or integrated molecular subtypes through unsupervised clustering of lesion-based profiles, identifying differences in hormone receptor expression, immune infiltration patterns, and fibrotic features [58,77,135,136]. A critical distinction is that these represent lesion-level rather than patient-level classifications; a single patient may harbor lesions of different molecular profiles, and it remains unknown which, if any, drives symptom trajectory [1,2,20,77]. Advancing subtyping toward prognostic utility would require patient-level integrated profiles (combining lesion, endometrial, and circulating features) validated against longitudinal outcomes such as pain progression, recurrence, and treatment response in independent cohorts.

#### 3.2.1. Prognostic Biomarkers for Disease Trajectory

Candidate biomarkers including circulating miRNAs, CA-125, and endometrial molecular features have been associated with disease severity or symptom intensity in cross-sectional designs [80,135,137,138]. Endometrial markers of progesterone resistance—PR-B/PR-A ratio, membrane PR expression, FOXO1, and HOXA10 methylation—are among the most mechanistically grounded candidates and are discussed in detail in Section 3.3 in the context of treatment-response prediction. For all candidates in this category, the shared developmental need is longitudinal cohort studies with serial biomarker measurements, standardized outcome definitions, and sufficient follow-up to distinguish prognostic signals from cross-sectional associations [2,27,57,90].

#### 3.2.2. Infertility Prognosis

Proposed biomarkers span multiple biological levels: endometrial receptivity signatures [135,141,145], ovarian reserve markers such as AMH [139,142], inflammatory mediators in peritoneal fluid [20], and lesion-based angiogenic factors [1]. These show associations with intermediate endpoints, such as implantation rates and fertilization success, in small retrospective studies, but none has demonstrated clinical utility for guiding fertility treatment decisions in prospective trials [135,140]. Infertility in endometriosis is inherently multifactorial, involving anatomical distortion, inflammatory microenvironments, and endometrial defects, which poses a fundamental challenge for any single-omics predictor [1,20,27,140]. Integrated clinico-molecular models, combining imaging-based anatomical assessment, circulating biomarkers, and endometrial profiling, may offer a more realistic framework for fertility prediction than isolated molecular signatures, but this architecture has not yet been tested prospectively.

Because the prognostic and treatment-response literature is heterogeneous and often reported as small cross-sectional, retrospective, or mechanistic cohorts rather than standardized longitudinal validation studies, Table 4 summarizes the approximate evidence scale and design class rather than exact pooled sample-size estimates. This structure allows candidate classes to be compared by marker class, specimen type, endpoint, follow-up status, evidence tier, key limitation, and supporting evidence while avoiding overinterpretation of underpowered or non-comparable studies.Across these prognostic and predictive applications, the evidence base remains consistently less mature than diagnostic biomarker research. Most available studies are cross-sectional, retrospective, mechanistic, or linked to intermediate endpoints rather than prospectively designed to predict patient-level outcomes. No biomarker class currently has independent, prospective, patient-level longitudinal validation demonstrating prediction of pain trajectory, recurrence, live birth, or treatment response. All prognostic and treatment-response applications therefore remain at Tier 1, despite the biological plausibility of several candidate markers. The most tractable near-term strategy is to embed candidate prognostic and predictive markers as companion analyses within ongoing or planned therapeutic trials, thereby generating paired biomarker–outcome data without requiring standalone biomarker studies.

### 3.3. Treatment-Response Prediction

Treatment-response prediction represents a distinct translational goal from diagnosis. A diagnostic biomarker asks whether endometriosis is present, whereas a predictive biomarker should identify, before treatment, which patients are more likely to benefit from a specific therapeutic strategy. In endometriosis, this is particularly relevant because empirical hormonal therapy, surgery, and emerging targeted treatments produce heterogeneous outcomes, and current treatment selection is still guided mainly by symptoms, lesion phenotype, fertility goals, contraindications, and patient preference rather than molecular stratification [26,29,36]. Despite strong biological rationale, no molecular marker has yet been validated prospectively to guide treatment allocation or improve patient-relevant outcomes compared with empirical care. For this reason, treatment-response applications currently remain Tier 1.

#### 3.3.1. Progestin Response and Progesterone Resistance

Progesterone resistance is one of the most biologically plausible domains for treatment-response prediction. A convergent molecular pattern has been described in eutopic endometrium and lesions, including reduced PR-B relative to PR-A, altered membrane progesterone receptor signaling, decreased expression of progesterone-regulated mediators such as FOXO1 and HOXA10, and epigenetic dysregulation of progesterone-responsive pathways [52,54,74,142,143,144]. These findings provide a mechanistic rationale for distinguishing patients who may respond poorly to progestin-based therapy from those in whom progesterone signaling remains functionally intact.

However, the translational evidence remains early. Most available data are mechanistic, cross-sectional, or based on tissue obtained at surgery rather than on prospective pretreatment biomarker assessment linked to standardized therapeutic outcomes [20,52,53,54,74]. A practical limitation is that assessment of endometrial or lesion-level progesterone signaling currently requires invasive sampling, and no validated blood-, urine-, saliva-, imaging-, or metabolite-based surrogate has been shown to reliably capture progesterone-resistance status in a way that can guide therapy [4,17,111]. Therefore, progesterone-resistance markers remain biologically compelling but clinically non-actionable.

A feasible next step would be to embed progesterone-pathway markers into prospective progestin-treatment cohorts or randomized trials as prespecified companion analyses. Such studies would need pretreatment sampling, standardized symptom and quality-of-life endpoints, defined treatment exposure and adherence, and follow-up sufficient to distinguish early symptom fluctuation from sustained response. Only if biomarker-defined strata predict differential benefit from progestin therapy, and if biomarker-guided treatment improves outcomes compared with empirical allocation, could this domain progress beyond Tier 1.

#### 3.3.2. Surgical Response Prediction

Surgical response prediction is clinically important because postoperative outcomes vary substantially. Some patients experience durable improvement, whereas others have persistent pain, recurrent symptoms, or lesion recurrence despite technically adequate surgery [35,36]. Candidate predictors include lesion fibrosis, nerve-fiber density, inflammatory-cell infiltration, angiogenic features, and other microenvironmental characteristics that may relate to pain generation, lesion persistence, or recurrence risk [35,36,49]. These features are biologically plausible because they reflect tissue remodeling, neuroangiogenesis, inflammation, and fibrosis, processes that are central to symptomatic disease.

The main translational barrier is timing. Most lesion-level features can only be measured after excision, which limits their usefulness for preoperative decision-making. As a result, current evidence can generate hypotheses about why some patients respond poorly to surgery, but it does not yet provide a validated tool for deciding before surgery who should undergo excision, who is at high risk of recurrence, or who requires intensified postoperative suppression [1,2,9,35]. In addition, many studies use retrospective designs, heterogeneous definitions of recurrence, and variable follow-up duration, making it difficult to separate molecular predictors from surgical completeness, lesion phenotype, postoperative treatment, or baseline pain mechanisms. Progress toward clinical utility would require prospective surgical cohorts with standardized preoperative phenotyping, detailed operative classification, biospecimen collection, and longitudinal follow-up for pain, quality of life, recurrence, and fertility outcomes. The most clinically useful model would likely combine molecular features with imaging, surgical phenotype, baseline pain characteristics, and postoperative treatment exposure rather than rely on a single lesion marker. Until such patient-level longitudinal validation exists, surgical-response biomarkers remain Tier 1.

#### 3.3.3. Emerging Targeted Therapies and the Case for Biomarker-Stratified Trials

Emerging therapeutic strategies provide another rationale for predictive biomarkers. Approaches targeting local estrogen production, gonadotropin signaling, inflammation, angiogenesis, fibrosis, or immune dysregulation are biologically plausible because endometriosis lesions show heterogeneity in steroid responsiveness, inflammatory signaling, vascular remodeling, and tissue fibrosis [1,26,35,36,51]. In principle, molecular profiling could help identify patients most likely to benefit from specific agents, for example, aromatase-related strategies in lesions with high estrogen-biosynthesis activity or anti-inflammatory approaches in patients with cytokine-enriched phenotypes. At present, however, this remains a translational hypothesis rather than a validated treatment-selection strategy [20,146]. Trials and therapeutic studies generally enroll molecularly unselected populations, which may dilute treatment effects if only a biologically defined subgroup is responsive [1,9,27]. Conversely, exploratory molecular subgroup findings cannot be assumed to predict treatment benefit unless they are tested prospectively with prespecified interaction analyses or biomarker-stratified allocation. This distinction is important: a marker associated with disease biology is not necessarily a predictive biomarker.

The most efficient path forward is not to create standalone predictive-biomarker studies, but to embed molecular profiling into therapeutic trials and prospective treatment cohorts. Even initially exploratory companion analyses could generate paired biomarker–outcome data, identify candidate responder subgroups, and inform future stratified trials. For any candidate predictive marker to progress beyond Tier 1, it would need to show that biomarker status modifies treatment effect and that biomarker-guided allocation improves patient-relevant outcomes compared with empirical treatment.

#### 3.3.4. Translational Takeaway

Treatment-response prediction in endometriosis remains less mature than diagnostic biomarker development. The strongest biological rationale exists for progesterone-resistance markers, lesion microenvironment features related to surgical outcomes, and molecular stratification of emerging targeted therapies. However, these candidates remain limited by invasive sampling, retrospective or mechanistic evidence, lack of standardized outcomes, and absence of prospective biomarker-stratified treatment studies. The most tractable near-term opportunity is to incorporate companion molecular profiling into planned progestin, surgical, and novel-agent studies, with patient-relevant endpoints such as pain, quality of life, fertility, recurrence, and treatment discontinuation. Until biomarker-defined groups are shown to predict differential treatment benefit, treatment-response applications should remain classified as Tier 1.

## 4. Multi-Omics Integration and Artificial Intelligence

### 4.1. Rationale and Theoretical Promise

Single-omics approaches have provided valuable mechanistic insights; however, as previously discussed, they have not individually achieved the diagnostic performance, prognostic discrimination, or treatment-response prediction required for clinical translation [58,147]. Multi-omics integration combines multiple molecular layers—genomics, epigenomics, transcriptomics, proteomics, and metabolomics—within the same biological samples to capture disease complexity more comprehensively [147,148,149,150]. The conceptual appeal is that endometriosis spans genetic susceptibility, epigenetic dysregulation, immune dysfunction, hormonal aberrations, and metabolic remodeling (as detailed in Section 2 and Section 3), and integrated frameworks could reveal cross-layer regulatory relationships and identify convergent biological nodes that are difficult to detect from any single data type alone, favoring these as mechanistically plausible biomarker candidates or intervention points [58,96,147,150].

### 4.2. What Integration Achieves, and What It Does Not

#### 4.2.1. What Integration Might Improve

Multi-omics integration strengthens mechanistic inference by cross-validating signals across biological layers [58,150]. For example, integrated analysis of GWAS and DNA methylation data has identified candidate regulatory genes such as ESR1 and PIK3CG in endometriotic lesions, providing stronger evidence for direct regulatory effects than either data type alone and helping distinguish causal routes from secondary transcriptional consequences [58,148,149,150]. Such convergence across layers can support pathway-based therapeutic targeting by identifying nodes where genetic, epigenetic, transcriptional, and proteomic alterations intersect.

#### 4.2.2. What Integration Does Not Improve

Despite enhanced biological coherence, multi-omics integration has not yet demonstrably optimized diagnostic, prognostic, or treatment-response prediction at the individual patient level compared with the best-performing single-layer approaches [95,96,97,151]. No published multi-omics study has shown that integrated non-invasive classifiers outperform expert TVUS for OMA and DIE [8], the salivary miRNA classifier reported in developer-led validation [65,84,151], or clinical risk-factor models for prognosis [43,151]. Most published multi-omics classifiers remain discovery-stage and report AUCs of approximately 0.70–0.85 in small or highly selected cohorts [87,152,153].

This limited predictive gain likely reflects methodological rather than purely biological constraints. Most endometriosis multi-omics studies combine high-dimensional data layers in cohorts that remain small relative to the number of candidate predictors, increasing the risk of overfitting and unstable feature selection [95,96,150]. Integration is also complicated by tissue and time-point mismatch, because lesion, eutopic endometrium, blood, saliva, urine, microbiome, and imaging features may reflect different biological compartments and temporal scales. Batch effects, platform heterogeneity, incomplete phenotype harmonization, and limited external validation further reduce transportability. Consequently, multi-omics integration can strengthen mechanistic coherence, but it has not yet demonstrated superior patient-level diagnostic, prognostic, or treatment-response prediction compared with the best single-layer approaches or contemporary imaging pathways.

*Domain-specific hurdles:* Beyond the field-wide constraints of small cohorts, case-control inflation, and absent external validation that apply across omics domains (see Section 2 and Section 3), multi-omics integration introduces the additional challenge of combining high-dimensional data from multiple platforms in samples that are often too small for stable model development. Most studies remain at the lesion level rather than the patient level, which limits applicability to non-invasive clinical contexts, and no study has anchored integrated profiles to clinically meaningful endpoints beyond case-control discrimination [96,150].

*Translational takeaway*: Multi-omics integration is valuable for biological discovery and generation of hypotheses but has not yet translated to superior clinical prediction. Before additional multi-omics discovery studies are prioritized, existing single-omics candidates with the strongest preliminary evidence warrant rigorous independent validation, as these represent the most efficient path to near-term clinical testing [50,95,147].

### 4.3. Artificial Intelligence and Machine Learning

AI and machine learning (ML) present opportunities to uncover complex, nonlinear patterns within high-dimensional omics data, potentially surpassing the capabilities of traditional statistical analyses [147,150,154,155]. In the study of endometriosis, AI has been applied in two primary areas: first, in the automated interpretation of TVUS and MRI images to standardize lesion detection and minimize operator variability [41,42,96,97]; and second, for multi-omics classification using methods like deep learning, random forests, or gradient boosting, which have achieved promising AUCs of 0.81–0.92 in initial discovery settings [63,156].

*Current evidence and limitations:* A consistent finding across comparative studies is that classical ML algorithms (random forests, support vector machines) perform comparably to, or outperform, deep neural networks when sample sizes are small, as overfitting risk increases with model complexity without proportional gains in generalizability [156,157]. AI’s advantage over simpler methods typically emerges with large datasets (*n* > 1000), a threshold rarely met in current endometriosis omics studies [63,150]. Additional concerns include model interpretability and the data requirements of deep learning architectures relative to available cohort sizes [150,156,157]. No AI-based omics classifier in endometriosis has undergone external validation or head-to-head comparison with simpler statistical approaches or expert imaging [63,96,97].

*Constructive future direction:* A promising but currently underexplored application is the integration of omics biomarkers with imaging data and clinical features within AI frameworks [97,158]. Rather than replacing imaging, such hybrid models could enhance detection of superficial peritoneal disease, where imaging performs poorly (see Table 2), refine risk stratification in imaging-ambiguous cases, or predict treatment response by combining molecular profiles with anatomical features. This architecture aligns with the adjunctive biomarker role but would require prospective validation against clinical utility endpoints, not just technical accuracy metrics.

*Translational takeaway:* AI offers methodological tools for pattern recognition but does not circumvent the requirements for adequate sample sizes, external validation, and clinical utility demonstration. Caution is warranted against “innovation theater”; deploying complex AI methods on small datasets that generate publishable accuracy metrics without advancing clinical translation.

## 5. From Evidence Gaps to Validation Architecture

The preceding sections reveal a consistent pattern: omics research in endometriosis has produced rich mechanistic biology but no candidate has crossed the threshold from internal validation to independent, clinically anchored evidence. Closing this gap requires more than better-powered replication; it requires matching candidates to the specific clinical problems they could realistically solve, with validation designs built around those problems rather than around the molecular data itself.

### 5.1. Matching Clinical Roles to Candidate Domains and Validation Requirements

Not all clinical needs are equally tractable, and not all omics domains are equally positioned to address them. The three diagnostic roles defined in Section 3.1—triage, adjunctive, and replacement—differ essentially in who is being tested, what decision the result informs, and what accuracy is required. The triage role has a defined candidate (salivary miRNA) and a feasible validation design, making it the most immediate priority for Tier 3 testing. The replacement role, while clinically appealing, lacks any candidate approaching the required thresholds and demands universal laparoscopy—a design that is increasingly difficult to justify ethically and logistically under contemporary guidelines that de-emphasize mandatory surgical diagnosis [9,31,35].

The adjunctive role occupies a strategically important middle ground. Expert imaging already performs well for OMA and DIE, but superficial peritoneal endometriosis remains a recognized diagnostic blind spot even in tertiary settings [8,35,44]. This is the clinical scenario where a molecular biomarker could provide information that imaging fundamentally cannot, detecting disease biology rather than anatomical distortion. An adjunctive biomarker validated in imaging-negative symptomatic women would have clinical value regardless of healthcare setting and would complement rather than compete with existing diagnostic pathways. Table 5 maps these distinctions to the omics candidates and validation architectures best suited to each role.

### 5.2. Validation Frameworks for Triage and Adjunctive Testing

**Figure 2:**
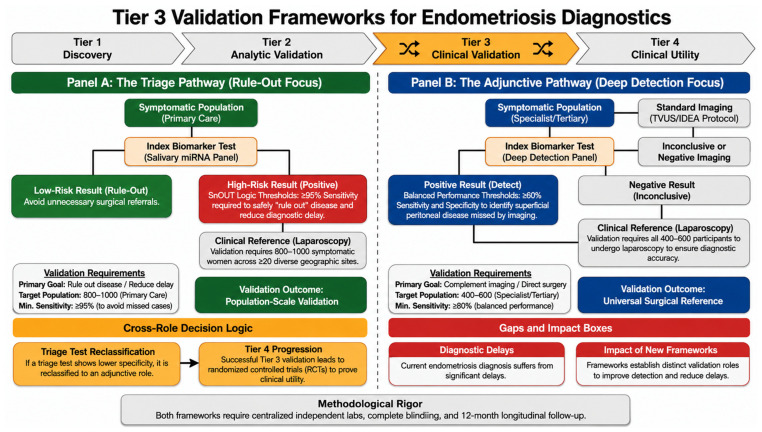
Proposed tier 3 validation frameworks for triage and adjunctive diagnostic testing in endometriosis. Two parallel validation pathways are presented for independent external validation of non-invasive omics-based biomarkers, positioned within the four-tier evidence-maturity framework (grey chevrons, top). Panel (**A**) (left, green header) illustrates a triage pathway with rule-out focus for the salivary miRNA panel in primary care, targeting ≥ 95% sensitivity (SnOUT logic) to sort symptomatic women into low-risk rule-out (red box) versus high-risk positive results requiring clinical reference by laparoscopy (green boxes). Validation requirements (white box, lower left). Panel (**B**) (right, blue header) illustrates a candidate-agnostic adjunctive pathway with deep detection focus for imaging-negative symptomatic women in specialist settings. Standard imaging by TVUS/IDEA protocol (grey box) identifies conclusive cases; inconclusive or negative imaging results (grey box) trigger the index biomarker test (orange box), targeting balanced performance (≥80% sensitivity and specificity) to detect superficial peritoneal disease missed by imaging (blue box). Universal laparoscopy serves as the clinical reference for all 400–600 enrolled participants (green box). Validation requirements (white box, lower right) specify balanced sensitivity thresholds in a specialist/tertiary population. The cross-role decision logic (bottom left) indicates that a triage test with lower specificity can be reclassified to an adjunctive role, and that successful Tier 3 validation leads to Tier 4 randomized controlled trials. The gaps and impact section (bottom right) summarizes current diagnostic delays and the expected impact of establishing distinct validation roles. Both frameworks share core methodological commitments (bottom banner): centralized independent laboratories, complete blinding, and 12-month longitudinal follow-up. Full operational specifications are provided in Box 2. Performance thresholds are derived from Section 3.1 and Table 5. Abbreviations: DIE, deep infiltrating endometriosis; IDEA, international deep endometriosis analysis; miRNA, microRNA; RCT, randomized controlled trial; SnOUT, sensitive test rules out; TVUS, transvaginal ultrasound.

Table 5 identifies triage and adjunctive testing as the two clinical roles with near-term translational tractability, while replacement testing is still premature, given the absence of candidates approaching the required thresholds as well as the ethical and logistical constraints of mandating universal laparoscopy in unselected populations [9,35,57]. This section provides operational validation frameworks for both roles (Box 2), designed to generate definitive Tier 3 evidence and serve as replicable templates for future omics candidates. The frameworks share core methodological commitments but differ in population, reference standard, and decision logic, differences that reflect the distinct clinical questions each role addresses; Figure 2 provides a visual overview of these two pathways.

Box 2 **Worked validation frameworks: triage and adjunctive testing.**

**Design Element**



**Triage Framework (Salivary miRNA)**



**Adjunctive Framework (Imaging-Negative Disease)**



**Candidate**

109-miRNA salivary panel (ENDOmiRNA) [62]Candidate-agnostic: proteomic, miRNA, microbiome, or metabolomic panels with ≥Tier 2 evidence

**Clinical question**

Empirical therapy vs. specialist referral in primary careDisease present despite negative expert imaging?

**Performance thresholds**

Sensitivity ≥ 95%, specificity ≥ 50% [24]Sensitivity and specificity both ≥ 80% [17,58]

**Target population**

Symptomatic women aged 18–45, no prior surgical diagnosis, primary care or general gynecology [62,66]Symptomatic women aged 18–45 with negative or indeterminate expert TVUS (IDEA protocol) [9,46]

**Exclusion criteria**

Pregnancy, known gynecologic malignancy, and other prespecified conditions affecting safety or diagnostic adjudication; hormonal-treatment status should be documented and analyzed in prespecified strata rather than used as a blanket exclusion in advanced validationAs triage, plus imaging-positive OMA or DIE (already diagnosed); hormonal-treatment status should be documented and analyzed in prespecified strata rather than used as a blanket exclusion in advanced validation [24,159]

**Expected prevalence**

35–45% in symptomatic clinic populations [24,159]25–40% in imaging-negative symptomatic populations [31]

**Target sample size**

800–1000 [62,159]400–600 [149,150]

**Reference standard**

Expert TVUS (all) ± selective laparoscopy for positive/indeterminate imaging or persistent symptoms [35,46]Universal laparoscopy with systematic peritoneal inspection and biopsy; histological confirmation required [9,24]

**Rationale for reference standard design**

Selective laparoscopy mirrors contemporary guidelines; ethical and feasible in large cohorts [35]Universal laparoscopy necessary because clinical question concerns disease that imaging cannot detect; verification bias would be unacceptable [24]

**Index test specimen and timing**

Saliva or other candidate-specific biospecimen collected using a prespecified protocol; cycle phase and hormonal-treatment status documented, with follicular-phase standardization used when feasible and sensitivity analyses prespecified [148,160]Blood, saliva, urine, and/or cervicovaginal sample, depending on the candidate assay; cycle phase and hormonal-treatment status documented, with follicular-phase standardization used when feasible and sensitivity analyses prespecified [63,105,160]

**Laboratory**

Centralized, independent from developer; inter-assay reproducibility documented [109,154]Centralized, independent from developer [3,159]

**Blinding**

Complete: biomarker results withheld until study completion [146,147]Complete: biomarker results withheld until study completion [146,147]

**Follow-up**

12 months with symptom assessment at 3, 6, 12 months [149,150]12 months with symptom assessment at 3, 6, 12 months [149,150]

**Outcome adjudication**

Blinded expert committee (3 specialists), consensus for discordant cases [155]Blinded expert committee, consensus for discordant cases [155]

**Primary outcomes**

Sensitivity, specificity, PPV, NPV with 95% CI [146,151]Sensitivity, specificity, PPV, NPV with 95% CI [146,151]

**Key secondary outcomes**

Stratified by phenotype (OMA, DIE, superficial); head-to-head vs. TVUS in imaging-ambiguous casesStratified by surgical phenotype; net reclassification improvement [161]; prognostic analysis of biomarker+/laparoscopy− cases at 12 months

**Statistical framework**

Preregistered; STARD-compliant; 80% power for sensitivity ≥95%, specificity ≥50%, precision ±3–4% [146]Preregistered; STARD-compliant; precision ±5% around 80% thresholds [151]

**If successful →**

Tier 4 RCT: biomarker-guided referral vs. standard care, patient-relevant outcomes (pain, quality of life, fertility)Tier 4 RCT: laparoscopy in biomarker+/imaging− women vs. continued empirical management

**If unsuccessful →**

Re-focus on subpopulations or reclassify to adjunctive role (see cross-role logic)Clarifies biological plausibility of non-invasive detection of superficial peritoneal disease**Note:** Both frameworks require multicenter enrollment with geographic and practice-setting diversity [62,152]. Analytical validity must be independently established before diagnostic evaluation begins. Rather than assuming universal exclusion of current hormonal treatment in advanced validation, protocols should prespecify how hormonal exposure and cycle phase will be documented, stratified, and analyzed [160]. Untreated or post-washout subcohorts may be included for sensitivity analyses when ethically and clinically feasible [14,84,113,162].

The triage framework evaluates whether a salivary miRNA panel (see Box 2 and Figure 2, panel A) can sort symptomatic women in primary care into those manageable with empirical therapy versus those requiring specialist referral and imaging, a decision node that currently relies on clinical judgment alone in settings without expert TVUS [4,35,87]. Because the test is evaluated against the full diagnostic pathway (imaging ± selective laparoscopy), the reference standard mirrors contemporary clinical practice and does not require universal surgery [35,43,44]. The performance priority is high sensitivity (≥95%) to minimize missed cases, accepting moderate specificity (≥50%), given that false positives result in specialist referral rather than harm [49].

The adjunctive framework (see Box 2 and Figure 2, panel B) addresses a fundamentally different question: among women whose expert imaging is already negative, can a molecular biomarker detect disease that imaging cannot visualize? This targets the recognized diagnostic blind spot for superficial peritoneal endometriosis, where imaging sensitivity remains poor regardless of operator expertise [8,35]. The post-imaging enrichment strategy, enrolling only imaging-negative women, is the defining design feature, ensuring that the study directly tests clinical value-add over existing pathways rather than overall accuracy in mixed populations [49,62]. Universal laparoscopy is required for all participants because the clinical question concerns disease that imaging has missed; without surgical ground truth, the reference standard would be circular [9,49]. Balanced sensitivity and specificity (both ≥ 80%) are required because a positive result must justify further intervention, typically laparoscopy, in women whose imaging is already negative [17,61,62].

Despite these structural differences, both frameworks are built on shared methodological commitments that address the recurring validation failures identified across Section 2, Section 3 and Section 4: centralized laboratory processing independent from the biomarker developer, to exclude optimistic bias and confirm analytical reproducibility across sites [2,49,87,163]; complete blinding of index test results from clinicians and reference standard assessors [163]; follicular-phase standardized specimen collection (cycle days 5–10) to control the menstrual-cycle variability that has confounded prior studies [66,69,160,164,165]; 12-month longitudinal follow-up capturing symptom trajectories beyond the initial diagnostic assessment; prespecified, publicly registered statistical analysis plans following STARD reporting guidelines; and multicenter enrollment with geographic and practice-setting diversity to ensure generalizability [7,62,84,152,159].

#### 5.2.1. Candidate Selection Logic

The triage framework is candidate-specific: the salivary 109-miRNA panel (ENDOmiRNA test) is selected as an example of the most advanced non-invasive omics candidate, with high developer-reported diagnostic performance but no independent non-developer evaluation, making it the highest-priority target for Tier 3 testing. The adjunctive framework is deliberately candidate-agnostic, applicable to any biomarker measurable in accessible specimens that has completed at least internal validation (Tier 2). This reflects the current landscape where no single adjunctive candidate has earned clear prioritization: blood-based multi-protein panels [61,87], circulating miRNA signatures [65,66,80,81], cervicovaginal microbiome profiles [127,166], and serum or urinary metabolomic signatures [88,125] all have preliminary signals but require the structured evaluation this framework provides.

#### 5.2.2. Decision Logic and Cross-Role Reclassification

**Figure 3:**
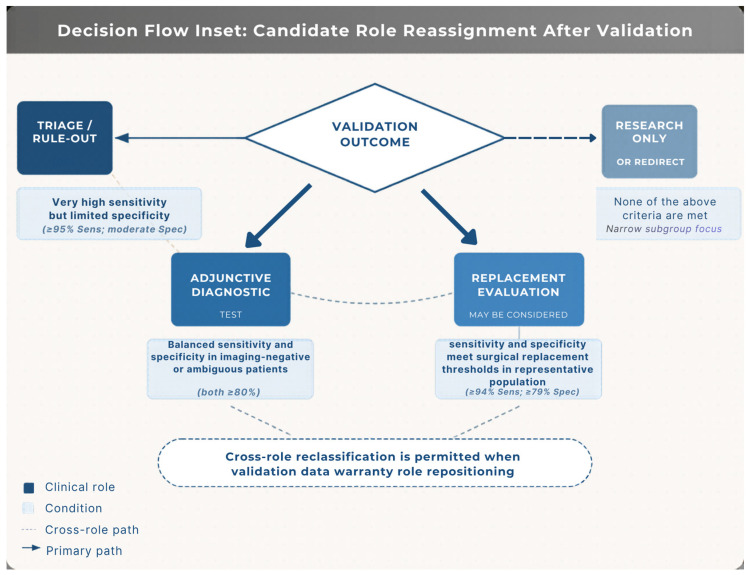
Decision-flow for biomarker role reassignment after validation. Candidate omics biomarkers should be interpreted according to their observed validation performance and intended clinical use context rather than treated as simple pass/fail entities. Very high sensitivity with limited specificity supports a triage or rule-out role. Balanced sensitivity and specificity in imaging-negative or imaging-ambiguous symptomatic patients supports an adjunctive diagnostic role. Performance meeting surgical replacement thresholds in a representative population with an appropriate surgical reference standard may justify subsequent replacement-test evaluation. Candidates that do not meet role-specific criteria should remain research-only or be redirected toward narrower biological or clinical subgroups.

A purposeful feature of presenting both frameworks together is that outcomes from one can inform the other. If the salivary miRNA triage test achieves high sensitivity but insufficient specificity, the adjunctive role, where moderate sensitivity is acceptable if balanced by adequate specificity, may represent a more appropriate clinical positioning than abandoning the candidate entirely. Conversely, if an adjunctive biomarker demonstrates unexpectedly high sensitivity across all phenotypes including imaging-positive disease, reclassification toward a triage or even replacement role could be considered, with appropriately designed subsequent studies [161]. This cross-role logic prevents the binary pass/fail interpretation that has characterized prior biomarker evaluation in endometriosis, where candidates were often discarded rather than repositioned when they failed to meet a single predetermined threshold [49,57]. Figure 3 summarizes the role-reassignment logic, and Box 2 provides the full operational specifications for the triage and adjunctive validation frameworks.

The sample-size ranges proposed in Box 2 should be interpreted as planning ranges for diagnostic-accuracy validation rather than definitive trial-power calculations. In diagnostic-test studies, sample size depends on expected disease prevalence, target sensitivity and specificity, acceptable confidence-interval width or lower confidence limit, anticipated missingness, and planned subgroup analyses. Final protocols should therefore perform formal diagnostic-accuracy sample-size calculations tailored to the candidate assay, intended clinical role, and validation setting [159,167,168].

### 5.3. Handling Hormonal and Cycle-Phase Confounding in Omics Validation

Hormonal treatment and menstrual cycle phase are recurrent sources of variability in endometriosis omics studies, particularly for circulating miRNAs, endometrial tissue markers, and other cycle-sensitive analytes [49,66,82,164,165,169]. Their handling should depend on the stage and purpose of biomarker development. In early discovery and analytical-validation studies, restricting sampling to a defined cycle phase and excluding current hormonal treatment may reduce biological noise and improve detectability of molecular signals. However, in advanced diagnostic validation and clinical utility studies, systematic exclusion of hormonally treated participants can substantially limit generalizability, because many symptomatic patients are already receiving hormonal therapy when diagnostic uncertainty arises [9,35,43].

A pragmatic validation strategy should therefore include hormonally treated participants in advanced validation cohorts when clinically relevant, document treatment class, duration, adherence, and time since last dose, and prespecify stratified and sensitivity analyses by hormonal-treatment status and cycle phase. For cycle-sensitive analytes, standardized sampling remains desirable when feasible, but real-world performance should also be evaluated under less restricted sampling conditions [66,82,83,160]. Conversely, if a candidate biomarker is proposed to be robust to hormonal treatment or cycle phase, this claim should be supported by explicit subgroup performance estimates and independent validation [66,160].

The salivary miRNA validation study provides an important example because saliva samples were collected irrespective of menstrual cycle phase and hormonal or analgesic treatment, and hormonally treated participants were included [84,164]. Nevertheless, unless separate diagnostic-performance estimates are available for treated and untreated participants, the claim should be phrased cautiously as “reported no apparent influence of hormonal-treatment status,” rather than as definitive equivalence across treatment strata.

## 6. Conclusions

### 6.1. Evidence-Based Summary

This translational assessment applied a four-tier evidence-maturity framework across omics domains and clinical applications in endometriosis. Despite a large and growing body of biomarker candidates spanning genomics, epigenomics, transcriptomics, proteomics, metabolomics, microbiome, extracellular vesicle, and multi-omics/AI platforms, no candidate has yet fulfilled Tier 3 (independent validation) or Tier 4 (clinical utility) criteria. Salivary miRNA represents the most advanced diagnostic candidate, with promising advanced Tier 2/Tier 2+ evidence from a multicenter prospective study [65,84], and Section 5 provides a worked validation framework for its progression to Tier 3. Prognostic, treatment-response, and multi-omics/AI applications remain at Tier 1, constrained by cross-sectional designs, small cohorts, and absence of patient-level longitudinal validation [2,20,96]. The gap between discovery volume and validation rigor is the central translational barrier in the field.

### 6.2. Implications for Clinical Practice

The current evidence supports omics biomarkers as research instruments that inform disease biology, trial design, and therapeutic target discovery, rather than as determinants of patient management. International guidelines recommend systematic symptom assessment and expert-guided TVUS as the diagnostic foundation, with laparoscopy reserved for imaging-ambiguous cases or when therapeutic surgical intervention is indicated [8,9]. No commercially available omics test has established the clinical validation necessary to alter guideline-based management. If patients provide commercial test results, it is suggested that clinicians interpret these as exploratory findings that lack demonstrated clinical validity and should not be used as a standalone basis to modify established treatment plans.

### 6.3. Priorities for the Field

The validation frameworks proposed in Section 5 define what rigorous Tier 3 evaluation requires for diagnostic applications. Beyond these specific designs, progression from the current discovery landscape towards clinically actionable tools will require strategic alignment across several dimensions.

*Population expansion*. The current evidence is overwhelmingly derived from European-ancestry cohorts of reproductive-age women in tertiary surgical settings [2,14,72]. Validation in understudied groups—adolescents, where early detection may slow disease progression [14,43]; women with imaging-negative mild disease, where diagnostic uncertainty is greatest [30,35]; and cohorts with diverse ancestry and geographic backgrounds [1,14]—is essential for establishing generalizability and addressing health equity gaps.

*Prognostic and predictive validation*. As we outlined in Section 3.2 and Section 3.3, the field lacks any prospective longitudinal study linking molecular profiles to pain trajectory, recurrence, or treatment response. The most tractable near-term strategy is embedding candidate prognostic and predictive markers as companion analyses within ongoing or planned therapeutic trials, generating paired biomarker–outcome data without requiring standalone biomarker studies [9,35].

*Infrastructure and standards*. Progress toward higher-tier validation would be accelerated by multicenter consortia that pair standardized phenotyping with harmonized sampling and shared biorepositories. Building on established infrastructures such as EPHect [98,170], these collaborations can help deliver the sample sizes and cross-site reproducibility that are difficult for individual centers to achieve consistently [12,57]. In parallel, routine alignment with STARD and TRIPOD reporting standards would make studies more interpretable and comparable across settings [6,7]; journals and funders can further support this by encouraging explicit reporting of validation tier in abstracts to strengthen evidence synthesis and reduce ambiguity in translational claims.

*Temporal expectations*. A pragmatic way to frame timelines is as a staged set of achievable milestones rather than fixed predictions. Over the near term, a reasonable objective is completion of Tier 3 independent validation for most advanced candidates, alongside initiation of adjunctive biomarker studies in imaging-negative populations. Over the medium term, the field can aim to advance successful candidates into Tier 4 clinical utility trials and to begin establishing prognostic tools through biomarker-stratified therapeutic trials. Over the longer term, integrating validated molecular profiles with imaging and clinical features for personalized detection, prognosis, and treatment selection remains a credible direction, but it is likely to depend on sustained prioritization of validation rigor, particularly independent replication and clinically anchored study designs, over exploratory novelty across the next decade. Until that evidentiary threshold is reached, clinical decision-making can continue to be anchored in evidence-based pathways reflected in contemporary international guidelines.

## Figures and Tables

**Table 1:** Comparison of this review with prior major published syntheses.

Feature ^1^	This Review	[4]	[1]	[2]
Explicit tier-based evidence-maturity framework	Yes	No	No	No
Benchmarking against imaging (TVUS/MRI)	Yes	No	No	Partial
Role-specific performance thresholds	Yes	No	No	No
Cross-domain tier synthesis	Yes	Partial	No	Yes
Worked Tier-3 validation example	Yes	No	No	No
Prognosis/treatment-response assessment	Yes	Limited	Yes	Limited

^1^ ‘Partial’ indicates that a feature is addressed incompletely or implicitly. TVUS: Transvaginal ultrasound; MRI: Magnetic resonance imaging.

**Table 2:** Diagnostic performance metrics of contemporary imaging for endometriosis.

Modality	Phenotype	Sensitivity ^1,3^	Specificity ^1,3^	Setting ^2^	Key Limitation
Expert TVUS [8,44]	OMA	0.89 (0.86–0.92)	0.95 (0.92–0.97)	Specialist	Operator-dependent
Expert TVUS [8,35,41]	DIE (any)	0.79 (0.76–0.83)	0.94 (0.88–0.97)	Specialist	Site-specific variation
Expert TVUS [8,35,45]	SUP	<0.30	≈0.95–1.00	All	Poor visualization
Specialist MRI [8,44]	OMA	0.94 (0.74–0.99)	0.94 (0.89–0.97)	Tertiary	Cost; availability
Specialist MRI [34,41,46]	DIE (bowel)	0.93 (0.87–0.96)	0.96 (0.92–0.98)	Tertiary	Bowel prep required
Specialist MRI [8,41,47]	SUP	0.40–0.60	0.85–0.90	Tertiary	Signal overlap

^1^ Performance estimates reflect expert operators with the use of standardized protocols and should be interpreted in the context of the reported referral spectrum. ^2^ “Specialist” denotes dedicated endometriosis imaging services using protocolized TVUS/MRI; “Tertiary” denotes tertiary referral centers with high-volume expertise and more complex case mix; “All” denotes pooled or mixed settings when the source synthesis was not restricted to tertiary referral centers. ^3^ For OMA and DIE, these values approximate Cochrane-derived replacement-test benchmarks (sensitivity ≥ 94%, specificity ≥ 79%) used for Tier 3/4 molecular test thresholds; no current imaging or biomarker strategy reaches such performance for SUP, where visualization remains limited. Abbreviations: OMA, ovarian endometrioma; DIE, deep infiltrating endometriosis; SUP, superficial peritoneal disease.

**Table 3:** Evidence maturity by omics domain and clinical application.

Domain	Application	Tier ^1^	Justification
Genomics (GWAS, PRS)	Diagnosis	1	Robust statistical replication across large multi-ancestry cohorts [14,70,71]. However, PRSs explain only <5% of disease variance [16,70] and show modest discrimination (AUC ~ 0.55–0.62) for individual risk [71,72]. GWAS replication demonstrates population-level associations, rather than individual clinical validity [15,71,72].
Epigenomics	Diagnosis	1	Primarily relies on small discovery cohorts (*n* < 100) and cross-sectional designs [73,74]. While mechanistically informative regarding steroid hormone response and tissue origin, clinical application remains premature due to cell-type heterogeneity and menstrual cycle influence [58,73,74,75]. Gaps include a lack of independent external validation and substantial concerns regarding cell-type heterogeneity in bulk tissue samples.
Transcriptomics—Lesion	Mechanistic	1	Exhibits minimal signature overlap (<10%) across studies [76]. Typically uses small surgical cohorts (median *n* = 20–50) [77]. Findings are limited to invasive tissue analysis and currently lack prospective validation for non-invasive use [58,78].
Transcriptomics—Blood	Diagnosis	1	Dominated by small cohorts and “two-gate” case-control designs (laparoscopy cases vs. healthy controls) that inflate accuracy estimates [78,79,80,81]. Shows minimal replication across independent groups and inadequate documentation of the menstrual cycle [58,82,83].
Transcriptomics—Salivary miRNA	Diagnosis (Triage)	Advanced Tier 2/Tier 2+	Most advanced candidate: validated in a multicenter study (*n* = 971) across 17 centers using a prospective, blinded design (diagnostic accuracy 96.6%) [84]. Limitations precluding Tier 3: work is developer-led with no independent replication, relies on high-prevalence surgical populations (77–80%), and lacks head-to-head comparison with expert imaging [66,84].
Proteomics—Plasma	Diagnosis	1–2	Most candidates are Tier 1 due to small scale and lack of external validation [17,49,85,86]. Vodolazkaia 4-protein panel qualifies as lower Tier 2 (single-center prospective validation, *n* = 233, but failed subsequent internal validation) [61,87]. CA-125 alone is insufficient for clinical use [9].
Metabolomics	Diagnosis	1	Characterized by small discovery cohorts (*n* < 100) and a lack of external validation in independent prospective populations. Cross-study replication is minimal (<15%) [1]. Findings are often phenotype-specific and confounded by diet and medication [18,88].
Extracellular Vesicles	Diagnosis	1	Gaps include minimal MISEV compliance, contamination by lipoproteins, and a notable absence of large-scale validation studies [89,90].
Gut Microbiome	Diagnosis	1	Currently limited to small cohorts with minimal reproducibility at the species level [91,92]. Profiles are extensively confounded by geography, diet, and antibiotics [91]. No validated diagnostic signatures currently exist [91,93].
Multi-Omics Integration	Diagnosis	1	Offers enhanced biological coherence but shows no demonstrated improvement in patient-level prediction compared to single-omics (AUC 0.70–0.85) [94]. Constrained by small cohorts (*n* = 50–150), lack of external validation [63] and a lesion-level focus unsuitable for blood-based tests [94].
AI/ML Classifiers	Diagnosis	1	Reports AUC 0.85–0.92 in small discovery cohorts, but faces a substantial overfitting risk without large datasets [95]. No independent external validation or proven superiority over simpler models or expert imaging [96,97].
All Domains	Prognosis	1	Evidence only consists of cross-sectional association [98]. No prospective longitudinal validation links molecular profiles to individual symptom trajectories, recurrence risk, or disease progression [2,27].
All Domains	Treatment Response	1	Mechanistic associations with progesterone resistance and surgical outcomes have been identified [52]. However, there are no biomarker-stratified randomized trials demonstrating improved clinical outcomes through biomarker-guided allocation [1,26,52].

^1^ Tier 3 requires independent (non-developer) replication; multicenter enrollment across at least three geographically and clinically distinct sites; prospective or prospectively specified cohort design with consecutive enrollment; disease prevalence reflecting real-world settings; head-to-head comparison with relevant standard diagnostic pathways (expert imaging); prespecified analysis plan; STARD/TRIPOD adherence. No omics domain currently meets all Tier 3 criteria for any application.

**Table 4:** Summary of prognostic and treatment-response biomarker evidence.

Application	Candidate Marker/Class	Specimen Type	Representative Evidence Base	Endpoint	Follow-Up	Evidence Tier	Key Limitation
Molecular subtyping	Transcriptomic, proteomic, or integrated lesion clusters, including immune-enriched, stroma-enriched, and hormone-sensitivity-associated profiles	Lesion tissue; eutopic endometrium; occasionally integrated tissue-level profiles	Cross-sectional surgical cohorts and lesion-level integrative subtype analyses; evidence remains mainly discovery-scale and not patient-level longitudinal [77,106,135,136]	Molecular subtype; phenotype correlation; hormone-sensitivity hypotheses	None/cross-sectional	Tier 1	Lesion-level classification; absence of patient-level longitudinal validation; possible cross-lesion heterogeneity within the same patient
Pain trajectory	Circulating miRNAs, CA-125, inflammatory mediators, nerve-fiber markers, and tissue inflammatory/fibrotic features	Blood/serum; peritoneal fluid; lesion tissue; excised surgical specimens	Cross-sectional or retrospective association studies linking candidate markers to pain severity, disease stage, or inflammatory features; no prospective serial biomarker validation [50,80,137,138]	Pain intensity; chronic pelvic pain phenotype; symptom severity	Usually absent, cross-sectional, or retrospective	Tier 1	Associations are not validated as patient-level predictors of future pain trajectory; pain is multifactorial and poorly aligned with lesion burden
Recurrence prediction	CA-125, inflammatory markers, fibrosis markers, nerve-fiber density, and lesion-level histological or molecular features	Serum; lesion tissue; excised surgical specimens	Small retrospective or correlative surgical cohorts with heterogeneous recurrence definitions and variable outcome assessment; no standardized prospective recurrence-prediction model [37,50,139]	Symptom recurrence; lesion recurrence; postoperative pain persistence	Variable; often retrospective or based on postsurgical follow-up	Tier 1	No standardized recurrence definition across studies; candidate features are often measurable only after surgery; no validated non-invasive preoperative predictor
Fertility prognosis	Endometrial receptivity signatures, AMH, peritoneal inflammatory mediators, angiogenic factors, and lesion-associated molecular features	Endometrium; serum; peritoneal fluid; lesion tissue	Heterogeneous retrospective or small prospective fertility-related studies; many assess intermediate fertility endpoints rather than live birth [135,139,140,141,142]	Implantation; fertilization; pregnancy; live birth	Variable; often linked to fertility-treatment cycles rather than long-term reproductive follow-up	Tier 1	Intermediate endpoints are frequently used instead of live birth; infertility is multifactorial; no biomarker-stratified prospective fertility trial has demonstrated clinical utility
Progestin response	PR isoforms, PR-B/PR-A ratio, membrane progesterone receptors, FOXO1, HOXA10 methylation, PR-target genes, and progesterone-resistance signatures	Endometrial biopsy; eutopic endometrium; lesion tissue	Mechanistic and correlative studies linking progesterone-resistance biology to endometriosis pathophysiology and treatment response; no biomarker-guided therapeutic trial [20,54,55,73,143,144]	Symptom response to progestins; progesterone resistance; treatment selection	Usually absent or not biomarker-stratified	Tier 1	Requires invasive tissue sampling; no validated blood-based surrogate; no prospective biomarker-stratified trial comparing guided versus empirical hormonal therapy
Surgical response	Fibrosis markers, nerve-fiber density, inflammatory-cell infiltration, and lesion microenvironment features	Excised lesions; surgical specimens; occasionally paired clinical/surgical data	Small retrospective or correlative studies evaluating lesion biology and postsurgical outcomes; no validated preoperative surrogate [36,37,50]	Postoperative pain improvement; symptom recurrence; lesion recurrence	Variable; often retrospective or postsurgical	Tier 1	Features are generally assessable only after excision; cannot guide preoperative decision-making without validated non-invasive surrogates; no prospective outcome validation

Approximate study scale refers to the dominant design and validation level of the cited evidence rather than pooled sample-size estimates. Exact sample sizes vary across heterogeneous studies and were not meta-analytically pooled in this narrative translational assessment. Across applications, the absence of independent, prospective, patient-level longitudinal validation is the main reason these candidates remain Tier 1.

**Table 5:** Role-specific validation frameworks for diagnostic omics tests in endometriosis.

Attribute	Triage Test	Adjunctive Test	Replacement Test
Clinical decision node	Primary care: empirical therapy vs. specialist referral	Specialist setting: refine diagnosis when imaging is negative or ambiguous	Avoid diagnostic laparoscopy in selected patients
Target population	Symptomatic women without prior diagnosis, primary care or general gynecology	Symptomatic women with negative or inconclusive expert TVUS/MRI	Symptomatic women with inconclusive imaging who would otherwise undergo laparoscopy
Performance priority	Very high sensitivity (≥95%), high NPV; moderate specificity acceptable (≥50%) [49,69]	Balanced sensitivity and specificity (both ≥ 80%) in imaging-negative subgroup [17,61]	Sensitivity ≥ 94%, specificity ≥ 79% in symptomatic populations [2,4,57]
Best-positioned omics candidates	Salivary miRNA panel (diagnostic accuracy 96.6% in developer-led multicenter validation) [84]	Blood-based multi-protein panels, circulating miRNA, or microbiome signatures, candidates with signal in accessible specimens and plausible sensitivity to superficial/peritoneal disease [17,18,61,87,88]	No current candidate approaches these thresholds in independent validation; premature to nominate [2,57]
Reference standard	Expert TVUS (all) ± selective laparoscopy [35,46]	Expert TVUS/MRI as comparator; laparoscopy required for confirmation in imaging-negative cases.	Laparoscopy with histological confirmation mandatory for all participants [49]
Minimum design requirements	Prospective multicenter cohort, symptomatic population (~40% prevalence), blinded evaluation, ≥800 participants [64,87]	Prospective enriched cohort of imaging-negative symptomatic women, laparoscopic verification, ≥300–500 participants [159]	Prospective consecutive cohort, universal laparoscopy, ≥1000 participants to achieve adequate precision at high thresholds [57,159]
Feasibility and near-term tractability	High, candidate exists, validation design specified (Section 5.2), primary care pathway defined	Moderate, multiple candidate domains, no single leading panel; requires enrichment strategy for imaging-negative subgroup	Low, no candidate near threshold; universal laparoscopy requirement limits recruitment and ethical acceptability
Key unresolved question	Does developer-reported accuracy hold in independent, lower-prevalence symptomatic cohorts?	Can any accessible biomarker detect superficial peritoneal disease that expert imaging misses?	Can any non-invasive test match laparoscopic-level accuracy across all phenotypes?

## Data Availability

No new data were created or analyzed in this study. Data sharing is not applicable to this article.

## Abbreviations

The following abbreviations are used in this manuscript:

EDRN	Early Detection Research Network
IOM	Institute of Medicine (now known as the National Academy of Medicine)
TRIPOD	Transparent Reporting of a multivariable prediction model for Individual Prognosis or Diagnosis
STARD	Standards for Reporting Diagnostic Accuracy Studies
AI	Artificial Intelligence
AUC	Area Under the Receiver Operating Characteristic Curve
DIE	Deep Infiltrating Endometriosis
ESHRE	European Society of Human Reproduction and Embryology
EV	Extracellular Vesicle
GC-MS	Gas Chromatography–Mass Spectrometry
GWAS	Genome-Wide Association Study
LC-MS	Liquid Chromatography–Mass Spectrometry
MISEV	Minimal Information for Studies of Extracellular Vesicles
miRNA	MicroRNA
ML	Machine Learning
MRI	Magnetic Resonance Imaging
NMR	Nuclear Magnetic Resonance
NPV	Negative Predictive Value
OMA	Ovarian Endometrioma
PPV	Positive Predictive Value
PRS	Polygenic Risk Score
rASRM	Revised American Society for Reproductive Medicine
SEC	Size-Exclusion Chromatography
SUP	Superficial Peritoneal Disease
TVUS	Transvaginal Ultrasound

## References

[1] Giudice L.C., Oskotsky T.T., Falako S., Opoku-Anane J., Sirota M. (2023). Endometriosis in the Era of Precision Medicine and Impact on Sexual and Reproductive Health across the Lifespan and in Diverse Populations. FASEB J..

[2] Brulport A., Bourdon M., Vaiman D., Drouet C., Pocate-Cheriet K., Bouzid K., Marcellin L., Santulli P., Abo C., Jeljeli M. (2024). An Integrated Multi-Tissue Approach for Endometriosis Candidate Biomarkers: A Systematic Review. Reprod. Biol. Endocrinol..

[3] Pepe M.S., Etzioni R., Feng Z., Potter J.D., Thompson M.L., Thornquist M., Winget M., Yasui Y. (2001). Phases of Biomarker Development for Early Detection of Cancer. J. Natl. Cancer Inst..

[4] Anastasiu C.V., Moga M.A., Elena Neculau A., Bălan A., Scârneciu I., Dragomir R.M., Dull A.-M., Chicea L.-M. (2020). Biomarkers for the Noninvasive Diagnosis of Endometriosis: State of the Art and Future Perspectives. Int. J. Mol. Sci..

[5] Micheel C.M., Nass S.J., Omenn G.S., Committee on the Review of Omics-Based Tests for Predicting Patient Outcomesin Clinical Trials, Board on Health Care Services; Board on Health Sciences Policy, Institute of Medicine (2012). Evolution of Translational Omics: Lessons Learned and the Path Forward.

[6] Moons K.G.M., Altman D.G., Reitsma J.B., Ioannidis J.P.A., Macaskill P., Steyerberg E.W., Vickers A.J., Ransohoff D.F., Collins G.S. (2015). Transparent Reporting of a Multivariable Prediction Model for Individual Prognosis or Diagnosis (TRIPOD): Explanation and Elaboration. Ann. Intern. Med..

[7] Bossuyt P.M., Reitsma J.B., Bruns D.E., Gatsonis C.A., Glasziou P.P., Irwig L., Lijmer J.G., Moher D., Rennie D., de Vet H.C.W. (2015). STARD 2015: An Updated List of Essential Items for Reporting Diagnostic Accuracy Studies. BMJ.

[8] Singh S.S., Allaire C., Al-Nourhji O., Bougie O., Bridge-Cook P., Duigenan S., Kroft J., Lemyre M., Leonardi M., Leyland N. (2024). Guideline No. 449: Diagnosis and Impact of Endometriosis—A Canadian Guideline. J. Obstet. Gynaecol. Can..

[9] Becker C.M., Bokor A., Heikinheimo O., Horne A., Jansen F., Kiesel L., King K., Kvaskoff M., Nap A., Petersen K. (2022). ESHRE Guideline: Endometriosis. Hum. Reprod. Open.

[10] Tian Z., Chang X.-H., Zhao Y., Zhu H.-L. (2020). Current Biomarkers for the Detection of Endometriosis. Chin. Med. J..

[11] Ioannidis J.P.A., Bossuyt P.M.M. (2017). Waste, Leaks, and Failures in the Biomarker Pipeline. Clin. Chem..

[12] Feng Z., Pepe M.S. (2020). Adding Rigor to Biomarker Evaluations-EDRN Experience. Cancer Epidemiol. Biomark. Prev..

[13] Navanandan N., Searns J., Ambroggio L. (2023). Method/ology of Phases of Biomarker Discovery. Hosp. Pediatr..

[14] Rahmioglu N., Mortlock S., Ghiasi M., Møller P.L., Stefansdottir L., Galarneau G., Turman C., Danning R., Law M.H., Sapkota Y. (2023). The Genetic Basis of Endometriosis and Comorbidity with Other Pain and Inflammatory Conditions. Nat. Genet..

[15] Cardoso J.V., Perini J.A., Machado D.E., Pinto R., Medeiros R. (2020). Systematic Review of Genome-Wide Association Studies on Susceptibility to Endometriosis. Eur. J. Obstet. Gynecol. Reprod. Biol..

[16] Lalami I., Abo C., Borghese B., Chapron C., Vaiman D. (2021). Genomics of Endometriosis: From Genome Wide Association Studies to Exome Sequencing. Int. J. Mol. Sci..

[17] Azeze G.G., Wu L., Alemu B.K., Lee W.F., Fung L.W.Y., Cheung E.C.W., Zhang T., Wang C.C. (2024). Proteomics Approach to Discovering Non-Invasive Diagnostic Biomarkers and Understanding the Pathogenesis of Endometriosis: A Systematic Review and Meta-Analysis. J. Transl. Med..

[18] Collie B., Troisi J., Lombardi M., Symes S., Richards S. (2025). The Current Applications of Metabolomics in Understanding Endometriosis: A Systematic Review. Metabolites.

[19] Salliss M.E., Farland L.V., Mahnert N.D., Herbst-Kralovetz M.M. (2021). The Role of Gut and Genital Microbiota and the Estrobolome in Endometriosis, Infertility and Chronic Pelvic Pain. Hum. Reprod. Update.

[20] Ou Y., Wang H., Zhou C., Chen Y., Lyu J., Feng M., Huang X. (2025). Endometriosis-Associated Infertility: Multi-Omics Insights into Pathogenesis and Precision Therapeutics. Front. Endocrinol..

[21] Zheng Y., Wagner P.D., Singal A.G., Hanash S.M., Srivastava S., Huang Y., Zhao Y.-Q., Chari S.T., Marquez G., Etizioni R. (2024). Designing Rigorous and Efficient Clinical Utility Studies for Early Detection Biomarkers. Cancer Epidemiol. Biomark. Prev..

[22] Collins G.S., de Groot J.A., Dutton S., Omar O., Shanyinde M., Tajar A., Voysey M., Wharton R., Yu L.-M., Moons K.G. (2014). External Validation of Multivariable Prediction Models: A Systematic Review of Methodological Conduct and Reporting. BMC Med. Res. Methodol..

[23] Debray T.P.A., Vergouwe Y., Koffijberg H., Nieboer D., Steyerberg E.W., Moons K.G.M. (2015). A New Framework to Enhance the Interpretation of External Validation Studies of Clinical Prediction Models. J. Clin. Epidemiol..

[24] Baethge C., Goldbeck-Wood S., Mertens S. (2019). SANRA-a Scale for the Quality Assessment of Narrative Review Articles. Res. Integr. Peer Rev..

[25] Kapoor R., Stratopoulou C.A., Dolmans M.-M. (2021). Pathogenesis of Endometriosis: New Insights into Prospective Therapies. Int. J. Mol. Sci..

[26] Taylor H.S., Kotlyar A.M., Flores V.A. (2021). Endometriosis Is a Chronic Systemic Disease: Clinical Challenges and Novel Innovations. Lancet.

[27] Zondervan K.T., Becker C.M., Missmer S.A. (2020). Endometriosis. N. Engl. J. Med..

[28] Horne A.W., Missmer S.A. (2022). Pathophysiology, Diagnosis, and Management of Endometriosis. BMJ.

[29] Di Spiezio Sardo A., Becker C.M., Renner S.P., Suvitie P.A., Tarriel J.E., Vannuccini S., Garcia Velasco J.A., Verguts J., Mercorio A. (2025). Management of Women with Endometriosis in the 21st Century. Curr. Opin. Obstet. Gynecol..

[30] Young S.L. (2024). Nonsurgical Approaches to the Diagnosis and Evaluation of Endometriosis. Fertil. Steril..

[31] Van Gestel H., Bafort C., Meuleman C., Tomassetti C., Vanhie A. (2024). The Prevalence of Endometriosis in Unexplained Infertility: A Systematic Review. Reprod. Biomed. Online.

[32] Moïse A., Dzeitova M., de Landsheere L., Nisolle M., Brichant G. (2025). Endometriosis and Infertility: Gynecological Examination Practical Guide. J. Clin. Med..

[33] Lee D., Kim S.K., Lee J.R., Jee B.C. (2020). Management of Endometriosis-Related Infertility: Considerations and Treatment Options. Clin. Exp. Reprod. Med..

[34] Irungu S., Mavrelos D., Worthington J., Blyuss O., Saridogan E., Timms J.F. (2019). Discovery of Non-Invasive Biomarkers for the Diagnosis of Endometriosis. Clin. Proteom..

[35] As-Sanie S., Mackenzie S.C., Morrison L., Schrepf A., Zondervan K.T., Horne A.W., Missmer S.A. (2025). Endometriosis: A Review. JAMA.

[36] Saunders P.T.K., Horne A.W. (2021). Endometriosis: Etiology, Pathobiology, and Therapeutic Prospects. Cell.

[37] Pascoal E., Wessels J.M., Aas-Eng M.K., Abrao M.S., Condous G., Jurkovic D., Espada M., Exacoustos C., Ferrero S., Guerriero S. (2022). Strengths and Limitations of Diagnostic Tools for Endometriosis and Relevance in Diagnostic Test Accuracy Research. Ultrasound Obstet. Gynecol..

[38] Foti P.V., Farina R., Palmucci S., Vizzini I.A.A., Libertini N., Coronella M., Spadola S., Caltabiano R., Iraci M., Basile A. (2018). Endometriosis: Clinical Features, MR Imaging Findings and Pathologic Correlation. Insights Imaging.

[39] Pirtea P., Vulliemoz N., de Ziegler D., Ayoubi J.M. (2022). Infertility Workup: Identifying Endometriosis. Fertil. Steril..

[40] Capraș R.-D., Badea I.C., Moldovan M., Gaia-Oltean A.I., Badea A.-F., Telecan T. (2025). The Diagnostic Performance of Transvaginal Ultrasound for Posterior Compartment Endometriosis Compared to Laparoscopic and Histopathological Findings: A Systematic Review. Healthcare.

[41] Avery J.C., Deslandes A., Freger S.M., Leonardi M., Lo G., Carneiro G., Condous G., Hull M.L., Imagendo Study Group (2024). Noninvasive Diagnostic Imaging for Endometriosis Part 1: A Systematic Review of Recent Developments in Ultrasound, Combination Imaging, and Artificial Intelligence. Fertil. Steril..

[42] Cosgriff L., Mukker A., Ford C., Tavcar J. (2026). Advances in Non-Invasive Diagnostic Tools for Endometriosis: A Narrative Review of the Past Ten Years. Int. J. Gynaecol. Obstet..

[43] Agarwal S.K., Chapron C., Giudice L.C., Laufer M.R., Leyland N., Missmer S.A., Singh S.S., Taylor H.S. (2019). Clinical Diagnosis of Endometriosis: A Call to Action. Am. J. Obstet. Gynecol..

[44] Kanti F.S., Gorak Savard R., Bergeron F., Zomahoun H.T.V., Netter A., Maheux-Lacroix S. (2024). Transvaginal Ultrasound and Magnetic Resonance Imaging in the Diagnosis of Endometrioma: A Systematic Review and Meta-Analysis of Diagnostic Test Accuracy Studies. J. Obstet. Gynaecol..

[45] Mick I., Freger S.M., Marien M., Gholiof M., Leonardi M. (2025). Diagnostic Accuracy of Transvaginal Ultrasonography for Endometriosis According to the International Deep Endometriosis Analysis Consensus. O G Open.

[46] Guerriero S., Condous G., van den Bosch T., Valentin L., Leone F.P.G., Van Schoubroeck D., Exacoustos C., Installé A.J.F., Martins W.P., Abrao M.S. (2016). Systematic Approach to Sonographic Evaluation of the Pelvis in Women with Suspected Endometriosis, Including Terms, Definitions and Measurements: A Consensus Opinion from the International Deep Endometriosis Analysis (IDEA) Group. Ultrasound Obstet. Gynecol..

[47] Daniilidis A., Grigoriadis G., Dalakoura D., D’Alterio M.N., Angioni S., Roman H. (2022). Transvaginal Ultrasound in the Diagnosis and Assessment of Endometriosis-An Overview: How, Why, and When. Diagnostics.

[48] Surrey E., Soliman A.M., Trenz H., Blauer-Peterson C., Sluis A. (2020). Impact of Endometriosis Diagnostic Delays on Healthcare Resource Utilization and Costs. Adv. Ther..

[49] Nisenblat V., Bossuyt P.M.M., Shaikh R., Farquhar C., Jordan V., Scheffers C.S., Mol B.W.J., Johnson N., Hull M.L. (2016). Blood Biomarkers for the Non-Invasive Diagnosis of Endometriosis. Cochrane Database Syst. Rev..

[50] Gupta D., Hull M.L., Fraser I., Miller L., Bossuyt P.M.M., Johnson N., Nisenblat V. (2016). Endometrial Biomarkers for the Non-Invasive Diagnosis of Endometriosis. Cochrane Database Syst. Rev..

[51] Koninckx P.R., Fernandes R., Ussia A., Schindler L., Wattiez A., Al-Suwaidi S., Amro B., Al-Maamari B., Hakim Z., Tahlak M. (2021). Pathogenesis Based Diagnosis and Treatment of Endometriosis. Front. Endocrinol..

[52] Flores V.A., Vanhie A., Dang T., Taylor H.S. (2018). Progesterone Receptor Status Predicts Response to Progestin Therapy in Endometriosis. J. Clin. Endocrinol. Metab..

[53] Reis F.M., Coutinho L.M., Vannuccini S., Batteux F., Chapron C., Petraglia F. (2020). Progesterone Receptor Ligands for the Treatment of Endometriosis: The Mechanisms behind Therapeutic Success and Failure. Hum. Reprod. Update.

[54] Zhang P., Wang G. (2023). Progesterone Resistance in Endometriosis: Current Evidence and Putative Mechanisms. Int. J. Mol. Sci..

[55] D’Hooghe T., Hummelshoj L. (2006). Multi-Disciplinary Centres/networks of Excellence for Endometriosis Management and Research: A Proposal. Hum. Reprod..

[56] Quesada J., Härmä K., Reid S., Rao T., Lo G., Yang N., Karia S., Lee E., Borok N. (2023). Endometriosis: A Multimodal Imaging Review. Eur. J. Radiol..

[57] Hudson Q.J., Perricos A., Wenzl R., Yotova I. (2020). Challenges in Uncovering Non-Invasive Biomarkers of Endometriosis. Exp. Biol. Med..

[58] Samare-Najaf M., Razavinasab S.A., Samareh A., Jamali N. (2024). Omics-Based Novel Strategies in the Diagnosis of Endometriosis. Crit. Rev. Clin. Lab. Sci..

[59] Kiesel L., Sourouni M. (2019). Diagnosis of Endometriosis in the 21st Century. Climacteric.

[60] Gibbons T., Rahmioglu N., Zondervan K.T., Becker C.M. (2024). Crimson Clues: Advancing Endometriosis Detection and Management with Novel Blood Biomarkers. Fertil. Steril..

[61] Vodolazkaia A., El-Aalamat Y., Popovic D., Mihalyi A., Bossuyt X., Kyama C.M., Fassbender A., Bokor A., Schols D., Huskens D. (2012). Evaluation of a Panel of 28 Biomarkers for the Non-Invasive Diagnosis of Endometriosis. Hum. Reprod..

[62] Schoeman E.M., Bringans S., Peters K., Casey T., Andronis C., Chen L., Duong M., Girling J.E., Healey M., Boughton B.A. (2025). Identification of Plasma Protein Biomarkers for Endometriosis and the Development of Statistical Models for Disease Diagnosis. Hum. Reprod..

[63] Sivajohan B., Elgendi M., Menon C., Allaire C., Yong P., Bedaiwy M.A. (2022). Clinical Use of Artificial Intelligence in Endometriosis: A Scoping Review. npj Digit. Med..

[64] Vouk K., Hevir N., Ribić-Pucelj M., Haarpaintner G., Scherb H., Osredkar J., Möller G., Prehn C., Rižner T.L., Adamski J. (2012). Discovery of Phosphatidylcholines and Sphingomyelins as Biomarkers for Ovarian Endometriosis. Hum. Reprod..

[65] Bendifallah S., Suisse S., Puchar A., Delbos L., Poilblanc M., Descamps P., Golfier F., Jornea L., Bouteiller D., Touboul C. (2022). Salivary MicroRNA Signature for Diagnosis of Endometriosis. J. Clin. Med..

[66] Vanhie A., Caron E., Vermeersch E., O D., Tomassetti C., Meuleman C., Mestdagh P., D’Hooghe T.M. (2024). Circulating microRNAs as Non-Invasive Biomarkers in Endometriosis Diagnosis-A Systematic Review. Biomedicines.

[67] de Araújo Medeiros Santos C.M., de Souza A.T.B., Neta A.P.R., Freire L.V.P., Sarmento A.C.A., de Medeiros K.S., Luchessi A.D., Cobucci R.N., Gonçalves A.K., de O. Crispim J.C. (2025). Exosomal MicroRNAs as Epigenetic Biomarkers for Endometriosis: A Systematic Review and Bioinformatics Analysis. Int. J. Mol. Sci..

[68] Erraji H., El Ghanmi A., Louanjli N., Benahmed M., El Mansouri F., Zarqaoui M., Ghazi B. (2025). Leveraging Epigenetic Aberrations in the Pathogenesis of Endometriosis: From DNA Methylation to Non-Coding RNAs. Front. Genet..

[69] Nisenblat V., Sharkey D.J., Wang Z., Evans S.F., Healey M., Ohlsson Teague E.M.C., Print C.G., Robertson S.A., Hull M.L. (2019). Plasma miRNAs Display Limited Potential as Diagnostic Tools for Endometriosis. J. Clin. Endocrinol. Metab..

[70] Sapkota Y., Steinthorsdottir V., Morris A.P., Fassbender A., Rahmioglu N., De Vivo I., Buring J.E., Zhang F., Edwards T.L., Jones S. (2017). Meta-Analysis Identifies Five Novel Loci Associated with Endometriosis Highlighting Key Genes Involved in Hormone Metabolism. Nat. Commun..

[71] Koller D., He J., Løkhammer S., Aranda S., Qiu D., Davtian D., Chen Q., Xu Z., Mao Z., Friligkou E. (2026). Multi-Ancestry Genome-Wide Association and Integrated Multi-Omics Analyses of Endometriosis and Its Clinical Manifestations. Nat. Genet..

[72] Svensson A., Garcia-Etxebarria K., Åkesson A., Borgfeldt C., Roth B., Ek M., D’Amato M., Ohlsson B. (2022). Applicability of Polygenic Risk Scores in Endometriosis Clinical Presentation. BMC Womens Health.

[73] Ducreux B., Patrat C., Firmin J., Ferreux L., Chapron C., Marcellin L., Parpex G., Bourdon M., Vaiman D., Santulli P. (2025). Systematic Review on the DNA Methylation Role in Endometriosis: Current Evidence and Perspectives. Clin. Epigenetics.

[74] Bedrick B.S., Courtright L., Zhang J., Snow M., Amendola I.L.S., Nylander E., Cayton-Vaught K., Segars J., Singh B. (2024). A Systematic Review of Epigenetics of Endometriosis. F S Rev..

[75] Retis-Resendiz A.M., González-García I.N., León-Juárez M., Camacho-Arroyo I., Cerbón M., Vázquez-Martínez E.R. (2021). The Role of Epigenetic Mechanisms in the Regulation of Gene Expression in the Cyclical Endometrium. Clin. Epigenetics.

[76] Vargas E., García-Moreno E., Aghajanova L., Salumets A., Horcajadas J.A., Esteban F.J., Altmäe S. (2022). The Mid-Secretory Endometrial Transcriptomic Landscape in Endometriosis: A Meta-Analysis. Hum. Reprod. Open.

[77] Marla S., Mortlock S., Heinosalo T., Poutanen M., Montgomery G.W., McKinnon B.D. (2023). Gene Expression Profiles Separate Endometriosis Lesion Subtypes and Indicate a Sensitivity of Endometrioma to Estrogen Suppressive Treatments through Elevated ESR2 Expression. BMC Med..

[78] Fassbender A., Verbeeck N., Börnigen D., Kyama C.M., Bokor A., Vodolazkaia A., Peeraer K., Tomassetti C., Meuleman C., Gevaert O. (2012). Combined mRNA Microarray and Proteomic Analysis of Eutopic Endometrium of Women with and Without Endometriosis. Hum. Reprod..

[79] Papari E., Noruzinia M., Kashani L., Foster W.G. (2020). Identification of Candidate microRNA Markers of Endometriosis with the Use of next-Generation Sequencing and Quantitative Real-Time Polymerase Chain Reaction. Fertil. Steril..

[80] Moustafa S., Burn M., Mamillapalli R., Nematian S., Flores V., Taylor H.S. (2020). Accurate Diagnosis of Endometriosis Using Serum microRNAs. Am. J. Obstet. Gynecol..

[81] Vanhie A., O D., Peterse D., Beckers A., Cuéllar A., Fassbender A., Meuleman C., Mestdagh P., D’Hooghe T. (2019). Plasma miRNAs as Biomarkers for Endometriosis. Hum. Reprod..

[82] Chung J., Rogers P.A. (2025). Improving Replication in Endometrial Omics: Understanding the Influence of the Menstrual Cycle. Int. J. Mol. Sci..

[83] Brady P., Yousif A., Sasamoto N., Vitonis A.F., Fendler W., Stawiski K., Hornstein M.D., Terry K.L., Elias K.M., Missmer S.A. (2024). Plasma microRNA Expression in Adolescents and Young Adults with Endometriosis: The Importance of Hormone Use. Front. Reprod. Health.

[84] Bendifallah S., Roman H., Suisse S., Spiers A., Petit E., Delbos L., Dabi Y., Touboul C., Dennis T., Merlot B. (2025). Validation of a Saliva Micro-RNA Signature for Endometriosis. NEJM Evid..

[85] Sasamoto N., Ngo L., Vitonis A.F., Dillon S.T., Sieberg C.B., Missmer S.A., Libermann T.A., Terry K.L. (2023). Plasma Proteomic Profiles of Pain Subtypes in Adolescents and Young Adults with Endometriosis. Hum. Reprod..

[86] Méar L., Com E., Fathallah K., Guillot L., Lavigne R., Guével B., Fauconnier A., Vialard F., Pineau C. (2022). The Eutopic Endometrium Proteome in Endometriosis Reveals Candidate Markers and Molecular Mechanisms of Physiopathology. Diagnostics.

[87] O D.F., Fassbender A., Van Bree R., Laenen A., Peterse D.P., Vanhie A., Waelkens E., D’Hooghe T.M. (2019). Technical Verification and Assessment of Independent Validation of Biomarker Models for Endometriosis. Biomed. Res. Int..

[88] Tokarz J., Adamski J., Rižner T.L. (2020). Metabolomics for Diagnosis and Prognosis of Uterine Diseases? A Systematic Review. J. Pers. Med..

[89] Ronsini C., Fumiento P., Iavarone I., Greco P.F., Cobellis L., De Franciscis P. (2023). Liquid Biopsy in Endometriosis: A Systematic Review. Int. J. Mol. Sci..

[90] Apostolov A., Mladenović D., Tilk K., Lõhmus A., Baev V., Yahubyan G., Sola-Leyva A., Bergamelli M., Görgens A., Zhao C. (2025). Multi-Omics Analysis of Uterine Fluid Extracellular Vesicles Reveals a Resemblance with Endometrial Tissue across the Menstrual Cycle: Biological and Translational Insights. Hum. Reprod. Open.

[91] Pérez-Prieto I., Vargas E., Salas-Espejo E., Lüll K., Canha-Gouveia A., Pérez L., Altmäe S. (2024). Gut microbiome in endometriosis: A cohort study on 1000 individuals. BMC Med..

[92] Yuanyue L., Dimei O., Ling L., Dongyan R., Xiaomei W. (2025). Association between Endometriosis and Gut Microbiota: Systematic Review and Meta-Analysis. Front. Microbiol..

[93] Riganelli L., Iebba V., Piccioni M., Illuminati I., Bonfiglio G., Neroni B., Calvo L., Gagliardi A., Levrero M., Merlino L. (2020). Structural Variations of Vaginal and Endometrial Microbiota: Hints on Female Infertility. Front. Cell Infect. Microbiol..

[94] Li C., Xu X., Zhao X., Du B. (2025). The Inconsistent Pathogenesis of Endometriosis and Adenomyosis: Insights from Endometrial Metabolome and Microbiome. mSystems.

[95] Bokulich N.A., Łaniewski P., Adamov A., Chase D.M., Caporaso J.G., Herbst-Kralovetz M.M. (2022). Multi-Omics Data Integration Reveals Metabolome as the Top Predictor of the Cervicovaginal Microenvironment. PLoS Comput. Biol..

[96] Dungate B., Tucker D.R., Goodwin E., Yong P.J. (2024). Assessing the Utility of Artificial Intelligence in Endometriosis: Promises and Pitfalls. Womens Health.

[97] AlSaad R., Farrell T., Elhenidy A., Albasha S., Thomas R. (2026). Artificial Intelligence in Endometriosis Imaging: A Scoping Review. AI.

[98] Vitonis A.F., Vincent K., Rahmioglu N., Fassbender A., Buck Louis G.M., Hummelshoj L., Giudice L.C., Stratton P., Adamson G.D., Becker C.M. (2014). World Endometriosis Research Foundation Endometriosis Phenome and Biobanking Harmonization Project: II. Clinical and Covariate Phenotype Data Collection in Endometriosis Research. Fertil. Steril..

[99] Kloeve-Mogensen K., Rohde P.D., Twisttmann S., Nygaard M., Koldby K.M., Steffensen R., Dahl C.M., Rytter D., Overgaard M.T., Forman A. (2021). Polygenic Risk Score Prediction for Endometriosis. Front. Reprod. Health.

[100] McGrath I.M., Montgomery G.W., Mortlock S., International Endometriosis Genetics Consortium (2023). Polygenic Risk Score Phenome-Wide Association Study Reveals an Association between Endometriosis and Testosterone. BMC Med..

[101] Lewis C.M., Vassos E. (2020). Polygenic Risk Scores: From Research Tools to Clinical Instruments. Genome Med..

[102] Wray N.R., Lin T., Austin J., McGrath J.J., Hickie I.B., Murray G.K., Visscher P.M. (2021). From Basic Science to Clinical Application of Polygenic Risk Scores: A Primer. JAMA Psychiatry.

[103] Thong L.Y., McRae A.F., Sirota M., Giudice L., Montgomery G.W., Mortlock S. (2025). Methylation Risk Score Modelling in Endometriosis: Evidence for Non-Genetic DNA Methylation Effects in a Case-Control Study. Int. J. Mol. Sci..

[104] Adamczyk M., Wender-Ozegowska E., Kedzia M. (2022). Epigenetic Factors in Eutopic Endometrium in Women with Endometriosis and Infertility. Int. J. Mol. Sci..

[105] Marquardt R.M., Tran D.N., Lessey B.A., Rahman M.S., Jeong J.-W. (2023). Epigenetic Dysregulation in Endometriosis: Implications for Pathophysiology and Therapeutics. Endocr. Rev..

[106] Poli-Neto O.B., Meola J., Rosa-E-Silva J.C., Tiezzi D. (2020). Transcriptome Meta-Analysis Reveals Differences of Immune Profile between Eutopic Endometrium from Stage I-II and III-IV Endometriosis Independently of Hormonal Milieu. Sci. Rep..

[107] Wang Y., Chen Y., Xiao Y., Ruan J., Tian Q., Cheng Q., Chang K., Yi X. (2023). Distinct Subtypes of Endometriosis Identified Based on Stromal-Immune Microenvironment and Gene Expression: Implications for Hormone Therapy. Front. Immunol..

[108] Walker E.R., McGrane M., Aplin J.D., Brison D.R., Ruane P.T. (2023). A Systematic Review of Transcriptomic Studies of the Human Endometrium Reveals Inconsistently Reported Differentially Expressed Genes. Reprod. Fertil..

[109] Fonseca M.A.S., Haro M., Wright K.N., Lin X., Abbasi F., Sun J., Hernandez L., Orr N.L., Hong J., Choi-Kuaea Y. (2023). Single-Cell Transcriptomic Analysis of Endometriosis. Nat. Genet..

[110] Chico-Sordo L., Ruiz-Martínez T., Toribio M., González-Martín R., Spagnolo E., Domínguez F., Hernández A., García-Velasco J.A. (2024). Identification of miR-30c-5p microRNA in Serum as a Candidate Biomarker to Diagnose Endometriosis. Int. J. Mol. Sci..

[111] Dolińska W., Draper H., Othman L., Thompson C., Girvan S., Cunningham K., Allen J., Rigby A., Phillips K., Guinn B.-A. (2023). Accuracy and Utility of Blood and Urine Biomarkers for the Noninvasive Diagnosis of Endometriosis: A Systematic Literature Review and Meta-Analysis. F S Rev..

[112] Ravaggi A., Bergamaschi C., Conforti J., Ciravolo G., Zanotti L., Fabricio A.S.C., Gion M., Cappelletto E., Leon A.E., Rossetti D.O. (2026). Serum miRNA-Based Diagnostic Models for Endometriosis: From Discovery to Validation. Hum. Reprod..

[113] Zafari N., Tarafdari A.M., Izadi P., Noruzinia M., Yekaninejad M.S., Bahramy A., Mohebalian A. (2021). A Panel of Plasma miRNAs 199b-3p, 224-5p and Let-7d-3p as Non-Invasive Diagnostic Biomarkers for Endometriosis. Reprod. Sci..

[114] Bendifallah S., Dabi Y., Suisse S., Jornea L., Bouteiller D., Touboul C., Puchar A., Daraï E. (2022). MicroRNome Analysis Generates a Blood-Based Signature for Endometriosis. Sci. Rep..

[115] Hirsch M., Duffy J., Davis C.J., Nieves Plana M., Khan K.S., International Collaboration to Harmonise Outcomes and Measures for Endometriosis (2016). Diagnostic Accuracy of Cancer Antigen 125 for Endometriosis: A Systematic Review and Meta-Analysis. BJOG.

[116] Sasamoto N., Ngo L.H., Vitonis A.F., Dillon S.T., Aziz M., Shafrir A.L., Missmer S.A., Libermann T.A., Terry K.L. (2025). Prospective Evaluation of Plasma Proteins in Relation to Surgical Endometriosis Diagnosis in the Nurses’ Health Study II. EBioMedicine.

[117] Dutta M., Singh B., Joshi M., Das D., Subramani E., Maan M., Jana S.K., Sharma U., Das S., Dasgupta S. (2018). Metabolomics Reveals Perturbations in Endometrium and Serum of Minimal and Mild Endometriosis. Sci. Rep..

[118] Angioni S., Congiu F., Vitale S.G., D’Alterio M.N., Noto A., Monni G., Santoru M.L., Fanos V., Murgia F., Atzori L. (2023). Gas Chromatography-Mass Spectrometry (GC-MS) Metabolites Analysis in Endometriosis Patients: A Prospective Observational Translational Study. J. Clin. Med..

[119] Adamyan L., Pivazyan L., Zarova E., Avetisyan J., Laevskaya A., Sarkisova A., Stepanian A. (2024). Metabolomic Biomarkers of Endometriosis: A Systematic Review. J. Endometr. Uterine Disord..

[120] Murgia F., Angioni S., D’Alterio M.N., Pirarba S., Noto A., Santoru M.L., Tronci L., Fanos V., Atzori L., Congiu F. (2021). Metabolic Profile of Patients with Severe Endometriosis: A Prospective Experimental Study. Reprod. Sci..

[121] Scheck S., Paterson E.S.J., Henry C.E. (2022). A Promising Future for Endometriosis Diagnosis and Therapy: Extracellular Vesicles—A Systematic Review. Reprod. Biol. Endocrinol..

[122] Khalaj K., Miller J.E., Lingegowda H., Fazleabas A.T., Young S.L., Lessey B.A., Koti M., Tayade C. (2019). Extracellular Vesicles from Endometriosis Patients Are Characterized by a Unique miRNA-lncRNA Signature. JCI Insight.

[123] Nazri H.M., Greaves E., Quenby S., Dragovic R., Tapmeier T.T., Becker C.M. (2023). The Role of Small Extracellular Vesicle-miRNAs in Endometriosis. Hum. Reprod..

[124] Baxter E., Nair S., West Z., Salomon C., Obermair A. (2025). Extracellular Vesicles as Biomarkers for Endometrial Cancer—A Systematic Review. Transl. Oncol..

[125] Maignien C., Santulli P., Kateb F., Caradeuc C., Marcellin L., Pocate-Cheriet K., Bourdon M., Chouzenoux S., Batteux F., Bertho G. (2020). Endometriosis Phenotypes Are Associated with Specific Serum Metabolic Profiles Determined by Proton-Nuclear Magnetic Resonance. Reprod. Biomed. Online.

[126] Colonetti T., Saggioratto M.C., Grande A.J., Colonetti L., Junior J.C.D., Ceretta L.B., Roever L., Silva F.R., da Rosa M.I. (2023). Gut and Vaginal Microbiota in the Endometriosis: Systematic Review and Meta-Analysis. Biomed. Res. Int..

[127] Ata B., Yildiz S., Turkgeldi E., Brocal V.P., Dinleyici E.C., Moya A., Urman B. (2019). The Endobiota Study: Comparison of Vaginal, Cervical and Gut Microbiota Between Women with Stage 3/4 Endometriosis and Healthy Controls. Sci. Rep..

[128] Svensson A., Brunkwall L., Roth B., Orho-Melander M., Ohlsson B. (2021). Associations Between Endometriosis and Gut Microbiota. Reprod. Sci..

[129] Shan J., Ni Z., Cheng W., Zhou L., Zhai D., Sun S., Yu C. (2021). Gut Microbiota Imbalance and Its Correlations with Hormone and Inflammatory Factors in Patients with Stage 3/4 Endometriosis. Arch. Gynecol. Obstet..

[130] Escorcia Mora P., Valbuena D., Diez-Juan A. (2025). The Role of the Gut Microbiota in Female Reproductive and Gynecological Health: Insights into Endometrial Signaling Pathways. Life.

[131] Tang F., Deng M., Xu C., Yang R., Ji X., Hao M., Wang Y., Tian M., Geng Y., Miao J. (2024). Unraveling the Microbial Puzzle: Exploring the Intricate Role of Gut Microbiota in Endometriosis Pathogenesis. Front. Cell Infect. Microbiol..

[132] Facciotti F., Di Stefano G., Maragno P., Ferraro C., Dridi D., Somigliana E., Viganò P., Vercellini P., Casalechi M. (2025). Microbiome Dysbiosis and Endometriosis: A Systematic Scoping Review of Current Literature and Knowledge Gaps. Hum. Reprod. Open.

[133] Jiang I., Yong P.J., Allaire C., Bedaiwy M.A. (2021). Intricate Connections between the Microbiota and Endometriosis. Int. J. Mol. Sci..

[134] Iavarone I., Greco P.F., La Verde M., Morlando M., Torella M., de Franciscis P., Ronsini C. (2023). Correlations between Gut Microbial Composition, Pathophysiological and Surgical Aspects in Endometriosis: A Review of the Literature. Medicina.

[135] Limam I., Abdelkarim M., Kacem-Berjeb K., Khrouf M., Feki A., Braham M., Chakroun N. (2025). Precision Therapeutic and Preventive Molecular Strategies for Endometriosis-Associated Infertility. Int. J. Mol. Sci..

[136] Jørgensen H., Fedorcsak P., Isaacson K., Tevonian E., Xiao A., Beste M., Qvigstad E., Lauffenburger D., Griffith L. (2022). Endometrial Cytokines in Patients with and without Endometriosis Evaluated for Infertility. Fertil. Steril..

[137] Shokrnejad-Namin T., Farzizadeh N., Najmi Z., Amoozgar M., Hariri A., Amini E., Khosravi A., Zarrabi A. (2026). Evaluating the Diagnostic Potential of CA-125 and miRNA Levels in Endometriosis: A Narrative Review. Int. J. Gynaecol. Obstet..

[138] Abd Alredha R.D., Yaseen B.R., Mohammed A.J., Salman S.A. (2025). Integrative Analysis of Inflammatory Biomarkers and Clinical Features in Endometriosis: Insights into Pain Severity and Diagnostic Utility. Anaesth. Pain Intensive Care.

[139] Nouri B., Talebian N., Kharaz L., Falah Tafti M. (2025). Diagnostic Value of Serum Cancer Antigen 125, Carcinoembryonic Antigen, Cancer Antigen 19-9, Anti Müllerian Hormone, White Blood Cell Count, Platelet Count, and Neutrophil to Lymphocyte Ratio in Endometriosis: A Retrospective Study. Int. J. Fertil. Steril..

[140] Koutalia N., Gkrozou F., Vatopoulou A., Lentzaris D., Skentou C., Paschopoulos M. (2024). Role of Molecular Biomarkers in Endometriosis-Related Infertility: A Narrative Review of the Literature. Cureus.

[141] Li J., Liu H., Lim J., Xing H., Chen Y., Yang S., Fu X. (2025). Molecular and Biological Markers for Assessing Endometrial Receptivity in Infertile Women: A Narrative Review. J. Int. Med. Res..

[142] Grigoriadis G., Daniilidis A., Pitsillidi A., Biyik I., Crestani A., Merlot B., Roman H. (2025). Evidence on Serum Anti-Müllerian Hormone Levels and Endometriosis Surgery. J. Clin. Med..

[143] Houshdaran S., Nezhat C.R., Vo K.C., Zelenko Z., Irwin J.C., Giudice L.C. (2016). Aberrant Endometrial DNA Methylome and Associated Gene Expression in Women with Endometriosis. Biol. Reprod..

[144] Velázquez-Hernández D.M., Vázquez-Martínez E.R., Cruz-Orozco O., Silvestri-Tomassoni J.R., Sánchez-Ramírez B., Olguín-Ortega A., Escobar-Ponce L.F., Rodríguez-Dorantes M., Camacho-Arroyo I. (2025). Membrane Progesterone Receptor Beta Regulates the Decidualization of Endometrial Stromal Cells in Women with Endometriosis. Int. J. Mol. Sci..

[145] Ordiyants I.M., Zyukina Z.V., Novginov D.S., Asatryan D.R. (2023). Modern Concepts O F Endometrial Receptivity in Endometriosis-Associated Infertility (analytical Review). Fundam. Clin. Med..

[146] Simancas-Racines D., Jiménez-Flores E., Montalvan M., Horowitz R., Araujo V., Reytor-González C. (2026). Endometriosis as a Systemic and Complex Disease: Toward Phenotype-Based Classification and Personalized Therapy. Int. J. Mol. Sci..

[147] Wu Y., Xie L. (2025). AI-Driven Multi-Omics Integration for Multi-Scale Predictive Modeling of Genotype-Environment-Phenotype Relationships. Comput. Struct. Biotechnol. J..

[148] Chen L., Yi Y., Nie J. (2025). Multiomic Insight into the Involvement of Cell Aging Related Genes in the Pathogenesis of Endometriosis. Sci. Rep..

[149] Lei L., Xu X., Gong C., Lin B., Li F. (2023). Integrated Analysis of Genome-Wide Gene Expression and DNA Methylation Profiles Reveals Candidate Genes in Ovary Endometriosis. Front. Endocrinol..

[150] Ballard J.L., Wang Z., Li W., Shen L., Long Q. (2024). Deep Learning-Based Approaches for Multi-Omics Data Integration and Analysis. BioData Min..

[151] Mittal S., Tong A., Young S., Jha P. (2025). Artificial Intelligence Applications in Endometriosis Imaging. Abdom. Radiol..

[152] Zhang B., Lv X., Li D., Zhang L., Ru Z., Ma Y. (2025). Diagnostic Accuracy of Machine Learning for Endometriosis: A Systematic Review and Meta-Analysis. Front. Endocrinol..

[153] Qin D., Zheng Y., Wang L., Lin Z., Yao Y., Fei W., Zheng C. (2025). Unraveling Shared Diagnostic Genes and Cellular Microenvironmental Changes in Endometriosis and Recurrent Implantation Failure through Multi-Omics Analysis. Sci. Rep..

[154] Wei L., Niraula D., Gates E.D.H., Fu J., Luo Y., Nyflot M.J., Bowen S.R., El Naqa I.M., Cui S. (2023). Artificial Intelligence (AI) and Machine Learning (ML) in Precision Oncology: A Review on Enhancing Discoverability through Multiomics Integration. Br. J. Radiol..

[155] Sone K., Toyohara Y., Taguchi A., Miyamoto Y., Tanikawa M., Uchino-Mori M., Iriyama T., Tsuruga T., Osuga Y. (2021). Application of Artificial Intelligence in Gynecologic Malignancies: A Review. J. Obstet. Gynaecol. Res..

[156] Zhang Y., Qin Q. (2025). Prospects and Challenges of Deep Learning in Gynecologic Malignancies. Front. Oncol..

[157] Alhamrani S.Q., Ball G.R., El-Sherif A.A., Ahmed S., Mousa N.O., Alghorayed S.A., Alatawi N.A., Ali A.M., Alqahtani F.A., Gabre R.M. (2025). Machine Learning for Multi-Omics Characterization of Blood Cancers: A Systematic Review. Cells.

[158] Paiboonborirak C., Abu-Rustum N.R., Wilailak S. (2025). Artificial Intelligence in the Diagnosis and Management of Gynecologic Cancer. Int. J. Gynaecol. Obstet..

[159] Bujang M.A. (2023). An Elaboration on Sample Size Planning for Performing a One-Sample Sensitivity and Specificity Analysis by Basing on Calculations on a Specified 95% Confidence Interval Width. Diagnostics.

[160] Devesa-Peiro A., Sebastian-Leon P., Pellicer A., Diaz-Gimeno P. (2021). Guidelines for Biomarker Discovery in Endometrium: Correcting for Menstrual Cycle Bias Reveals New Genes Associated with Uterine Disorders. Mol. Hum. Reprod..

[161] Pencina M.J., D’Agostino R.B., Steyerberg E.W. (2011). Extensions of Net Reclassification Improvement Calculations to Measure Usefulness of New Biomarkers. Stat. Med..

[162] Buderer N.M. (1996). Statistical Methodology: I. Incorporating the Prevalence of Disease into the Sample Size Calculation for Sensitivity and Specificity. Acad. Emerg. Med..

[163] Christensen R., Ranstam J., Overgaard S., Wagner P. (2023). Guidelines for a Structured Manuscript: Statistical Methods and Reporting in Biomedical Research Journals. Acta Orthop..

[164] Mortazavi H., Yousefi-Koma A.-A., Yousefi-Koma H. (2024). Extensive Comparison of Salivary Collection, Transportation, Preparation, and Storage Methods: A Systematic Review. BMC Oral Health.

[165] d’Amone L., Matzeu G., Omenetto F.G. (2021). Stabilization of Salivary Biomarkers. ACS Biomater. Sci. Eng..

[166] Perrotta A.R., Borrelli G.M., Martins C.O., Kallas E.G., Sanabani S.S., Griffith L.G., Alm E.J., Abrao M.S. (2020). The Vaginal Microbiome as a Tool to Predict rASRM Stage of Disease in Endometriosis: A Pilot Study. Reprod. Sci..

[167] Flahault A., Cadilhac M., Thomas G. (2005). Sample Size Calculation Should Be Performed for Design Accuracy in Diagnostic Test Studies. J. Clin. Epidemiol..

[168] Bujang M.A., Adnan T.H. (2016). Requirements for Minimum Sample Size for Sensitivity and Specificity Analysis. J. Clin. Diagn. Res..

[169] Leonova A., Turpin V.E., Agarwal S.K., Leonardi M., Foster W.G. (2021). A Critical Appraisal of the Circulating Levels of Differentially Expressed microRNA in Endometriosis†. Biol. Reprod..

[170] Rahmioglu N., Fassbender A., Vitonis A.F., Tworoger S.S., Hummelshoj L., D’Hooghe T.M., Adamson G.D., Giudice L.C., Becker C.M., Zondervan K.T. (2014). World Endometriosis Research Foundation Endometriosis Phenome and Biobanking Harmonization Project: III. Fluid Biospecimen Collection, Processing, and Storage in Endometriosis Research. Fertil. Steril..

